# Mobility and Policy Responses During the COVID-19 Pandemic in 2020

**DOI:** 10.3389/ijph.2022.1604663

**Published:** 2022-08-05

**Authors:** Gabriel Cepaluni, Michael T. Dorsch, Daniel Kovarek

**Affiliations:** ^1^ Department of International Relations, São Paulo State University (UNESP), Franca, Brazil; ^2^ Department of Public Policy, Central European University (CEU), Vienna, Austria; ^3^ Democracy Institute, Central European University (CEU), Budapest, Hungary; ^4^ Department of Political Science, Central European University (CEU), Vienna, Austria

**Keywords:** COVID-19, geographic mobility, policy responses, instrumental variables, high-frequency panel data

## Abstract

**Objective:** This paper quantitatively explores determinants of governments’ non-pharmaceutical policy responses to the COVID-19 pandemic. Our focus is on the extent to which geographic mobility affected the stringency of governmental policy responses.

**Methods:** Using cross-country, daily frequency data on geographic mobility and COVID-19 policy stringency during 2020, we investigate some of the determinants of policy responses to COVID-19. In order to causally identify the effect of geographic mobility on policy stringency, we pursue an instrumental variable strategy that exploits climate data to identify arguably exogenous variation in geographic mobility.

**Results:** We find that societies that are more geographically mobile have governmental policy responses that are less stringent. Examining disaggregated mobility data, we show that the negative relation between geographic mobility and policy stringency is the stronger for commercially-oriented movements than for geographic movements that relate to civil society.

**Conclusion:** The results suggest that policy-makers are more willing to trade-off public health for economic concerns relative to other civil concerns.

## Introduction

In 2020 policy-makers around the world grappled with how to slow the spread of the novel coronavirus through their populations. There has been heterogeneity across countries and over time as to the stringency of policy responses and their effects, which has been the subject of much recent research in the social sciences (e.g., [1–4]). This paper contributes to the research on the determinants of country-level policy responses to the COVID-19 pandemic. We examine one specific factor, geographic mobility, that may influence the stringency of policy responses. Using daily variation in country-level mobility data from smartphones, we demonstrate a robust negative correlation between geographic mobility and policy stringency in a country-day panel regression analysis (see review of similar literature by [5]). An instrumental variable strategy allows us to identify the causal impact of geographic mobility.

Our explanation for the result is that the implementation of stringent policies is costlier (economically and politically) in more geographically mobile societies. Further analysis suggests that business interests play a larger role in limiting the intensity of governments’ policy responses than other civil society interests. An Supplementary Appendix provides an extensive robustness analysis of our main results as well as some further analysis. The last section before the conclusion details its contents. Our results have broader implications for preventing and controlling pandemics. After all, many times, mobile societies are those most in need to restrict people’s movements. An important contribution from social scientists is to identify and better understand the constraints on policy-makers.

## Methods

### Overview of Data

Our study employs country-level data over a sample of non-advanced economies (according to the International Monetary Fund classification) over the period of 22nd January 2020 until 31 August 2020. The countries are listed in Supplementary Table SA1. Our principal variables are high frequency, varying daily, while most of the control variables are fixed over the period of analysis.

Our main dependent variable is the Stringency Index of the Oxford COVID-19 Government Response Tracker (OxCGRT), which aggregates governmental responses over 17 distinct policy areas [6] for 197 countries and territories since the beginning of 2020. We also consider the disaggregated policy measures implemented by countries in a further analysis.

The main explanatory variable of interest is geographic mobility. Mobility Trend Reports (MTR) summarize requests for directions in Apple Maps, whereas Community Mobility Reports (CMR) include aggregated mobility statistics from Google Maps. Note that there is no one-to-one correspondence between using an Apple/Google device and having one’s movement recorded as data point in MTR/CMR. For instance, one can use Google Maps when planning a journey on an iPhone. In our analysis, we primarily rely on mobility data from Google, as it has better coverage for non-advanced economies and also allows us to disentangle mobility trends related to leisure, commercial or business purposes, or staying at home. CMR data shows the relative change in number of visitors (or time spent in) places categorized as supermarkets and pharmacies, parks, public transport, retail and recreation, residential areas and workplaces, compared to baseline days. These broad place categories are results of Google grouping multiple places with similar characteristics, such as public gardens and camp grounds for parks. Google uses a 5-week period immediately before the widespread disruption caused by COVID-19; the median value between 3 January–6 February 2020 serves as the baseline day in CMR. The dataset has been widely used by public health, social science and economics scholars to capture movement (see Supplementary Table SA.21 for related literature).

In our baseline specification, we lag the mobility data by its seven-day moving average, as we do not expect mobility to have instantaneous impact on policy decisions. In distinguishing between mobility related to the economy from that related to civil society, we make use of the COVID-19 Disorder Tracker, a curated selection of the Armed Conflict Location and Event Dataset (ACLED). It provides daily frequency data on political violence and protests related to COVID-19, with a coverage of 150 countries.

Our strategy for identifying the causal effect of geographic mobility employs precipitation as an instrument for mobility. Data on rainfall comes from the Global Surface Summary of the Day (GSOD) database, collected by the National Oceanic and Atmospheric Administration (NOAA). We compute daily average values for each country by adding up reported observations of precipitation levels for each country-day dyad and dividing them by the number of weather stations within a particular country. Following [7], we create a binary variable, rainy day, that takes value 1 with rainfall higher than 0.10 inch and 0 otherwise; we then subsequently calculate their 7-day moving averages.

We employ several control variables in our analysis: an indicator of quality of government, the level of democracy, which is an average of Freedom House and Polity indicators from the Quality of Government dataset, the (log of the) number of confirmed COVID-19 cases, the (log of) real GDP per capita, population density, the share of the elderly within the population, the share of trade as percentage of GDP, the number of hospital beds per 1,000 people, experience with the SARS epidemic in 2002–2003, as well as the (log of the) number of airports in the country. Besides the COVID-19 cases, these controls are repeated daily observations from data measured in 2019. Supplementary Section SA.1 describes the reasons for including these particular controls in our analysis.

Supplementary Figure SA.1 shows world maps of the incidence of our two principal mobility variables and the policy stringency index. Supplementary Tables SA.1–SA.3, SA.17–SA.19 provide summaries of the data and the samples used in the analysis, as well as a description of our data sources.

### Quantitative Methods

Our analysis employs regression techniques. First, we utilize the standard Ordinary Least Squares (OLS) regression with world regional and period (day) fixed effects.

$$Policy\text{\_}stringency_{i,j,t}=\alpha Mobility_{i,j,t-1}+X_{i,j}^{\prime }\beta +\gamma_{j}+\delta_{t}+u_{i,j,t},$$ (1)

where *Mobility*_*i*,*j*,*t*−1_ is the measure of geographic mobility that was described above for country *i* of region *j* on day *t* − 1. The *γ*_*j*_’s denote regional dummies that capture any time-invariant regional characteristics that affect countries’ policy responses and the *δ*_*t*_’s denote day dummies that capture common shocks to policy stringency levels. The vector **X**_*i*,*j*_ includes the battery of controls described in the previous section. The error term *u*_*i*,*j*,*t*_ captures all other factors not correlated with our controls which may also explain policy stringency, with *E*(*u*_*i*,*j*,*t*_) = 0 for all *i*, *j*, and *t*. All models include Driscoll-Kraay standard errors that are robust to cross-sectional and temporal dependences and autocorrelated consistent. Following [4], in some specifications, we also control for policy “adoption density,” which is calculated as the neighborhood average of the policy stringency index. For country *i* in a region *j* with *K* countries in period *t*, it is calculated as follows:

$$Adoption\text{\_}density_{i,j,t}=\frac{1}{K-1}\overset{K}{\underset{k\neq i}{\sum }}Policy\text{\_}stringency_{k,j,t}$$ (2)

The policy adoption density variable adds a powerful time-varying control.

One concern with estimating such a specification with OLS is that geographic mobility, even when lagged, may be endogenously determined. Such a concern would introduce a bias into our estimation of *α* and preclude a causal interpretation of the estimated results. We therefore pursue an instrumental variable identification strategy, in which we identify exogenous variation in lagged geographic mobility using lagged rainfall data. Rainfall is well-suited to instrumenting for geographic movement because it is intuitive that people stay more at home and walk less during rainy days. References [8, 9] review the literature that examines the direct impacts of weather variation on economic outcomes and political conflict, respectively. References [7, 10–12] use rainfall as an instrumental variable for political instabilities.

In our baseline specification, we use the binary rainy day variable as the instrument. Employing a Two-Stage Least Squares (TSLS) estimation procedure, in the first stage we estimate the following:

$$Mobility_{i,j,t-1}=\eta Rainy_{i,j,t-1}+X_{i,j}^{\prime }\theta +\gamma_{j}+\delta_{t}+e_{i,j,t},$$ (3)

We use the fitted values from Eq. 3 to estimate the impact of exogenous variation in geographic mobility on policy stringency in the second stage:

$$Policy\text{\_}stringency_{i,j,t}=\alpha^{2S}{\hat{Mobility}}_{i,j,t-1}+X_{i,j}^{\prime }\beta +\gamma_{j}+\delta_{t}+u_{i,j,t}.$$ (4)

In order for the estimation of *α* ^2*S*^ to provide a credible causal estimate of the impact of mobility on policy stringency, the rainfall instrument must satisfy two criteria: relevance and the exclusion restriction. First, the instrument should be strongly correlated with the main independent variable. The economic literature conventionally uses a first-stage F-statistic greater than 10 to indicate a strong instrument. The second condition requires that the instrument’s effect on the outcome variable occurs only through its influence on the potentially endogenous variable. As this assumption is not verifiable, we appeal to the intuition that rainfall affects mobility, but it does not directly affect governmental policy responses against the COVID-19 pandemic. To guard against possible exclusion restriction violations, we also employ a rich set of control variables, robustness checks, and a sensitivity analysis.

## Results

More geographical movement leads to less stringent policy responses. An analysis with valid instruments lends a causal interpretation of the relation, and the magnitude of the effect is strongest for movements related to economic and commercial activities.

### Ordinary Least Squares Results

First, Table 1 presents results from estimation of Eq. 1 with OLS. In columns (1)–(3) we use the Google data on staying put in residential areas, while in columns (4) and (5) we use the Apple data on walking.

**TABLE 1:** OLS regressions—Stringency index (Worldwide, 2020).

	(1)	(2)	(3)	(4)	(5)
Dependent variable: Stringency index					
Residential (7-day moving averages)	2.029*** (0.109)	1.567*** (0.193)	1.560*** (0.192)		
Walking (7-day moving averages)				−0.059** (0.021)	−0.055** (0.020)
Level of Democracy (Freedom House/Imputed Polity)		1.529+ (0.817)	1.624* (0.794)	−3.216*** (0.752)	−3.240*** (0.741)
ICRG Indicator of Quality of Government		−39.004*** (10.685)	−40.432*** (10.678)	56.498*** (16.842)	57.481*** (16.546)
Log (Confirmed cases)		0.237 (0.847)	0.299 (0.813)	3.808*** (0.722)	3.847*** (0.758)
Log (Real GDP per capita)		1.327 (2.741)	1.535 (2.646)	−13.971** (4.565)	−14.272** (4.620)
Population density (people per sq. km of land area)		0.006+ (0.003)	0.006+ (0.003)	0.028 (0.021)	0.028 (0.021)
Population ages 65 and above (% of total population)		−1.175+ (0.694)	−1.238+ (0.676)	−1.928** (0.675)	−1.970** (0.671)
Trade (% of GDP)		−0.100 (0.061)	−0.090 (0.059)	−0.084* (0.039)	−0.086* (0.040)
Hospital beds (per 1,000 people)		−0.648 (1.961)	−0.693 (1.955)	2.603 (2.469)	2.748 (2.478)
SARS		0.600** (0.183)	0.596*** (0.179)	−0.246 (0.171)	−0.255 (0.173)
Log (Airports)		1.600+ (0.928)	1.597+ (0.881)	−3.838* (1.857)	−3.983* (1.854)
Adoption density			−0.014 (0.066)		0.149** (0.055)
Historical rainfall			3.027 (2.281)		3.445 (2.796)
R-squared	0.534	0.430	0.438	0.441	0.451
Country-days	93	69	69	26	26
N	16,907	11,720	11,535	4,574	4,562
Region fixed effects		✓	✓	✓	✓
Day fixed effects		✓	✓	✓	✓

Notes: All specifications include Driscoll-Kraay (DK) standard errors (in parenthesis). DK non-parametric standard errors are heteroskedasticity robust to cross-country and day dependences and autocorrelated consistent (up to three-day lags). ^+^ *p* < 0.1, *p < 0.05, **p < 0.01, ***p < 0.001.

The first column of Table 1 presents the raw bivariate correlation between residential mobility and the stringency index. The positive coefficient on the residential mobility variable indicates that as countries become more residential compared to the baseline of January 2020, the stringency of responses has tended to increase. Using the summary statistics for the sample (see Supplementary Table SA.2), we calculate that one standard deviation increase in residential movement is associated with an 0.58 standard deviation increase in the policy stringency index. This result is confirmed as we add a battery of controls to the specification in column (2). Our most-preferred battery of controls follows from previous work on COVID-19 policy responses [1, 4]. First, we control for a measure of COVID-19 incidence, using daily data on confirmed cases (which we also lag using a seven-day moving average). We additionally include some time-invariant country-level controls for political institutions, state capacity, economic development, population density and demographics, economic globalization, experience with the SARS pandemic, and health care capacity. The coefficient on residential movement is estimated to be somewhat smaller when we include the battery of controls, but it remains positive and statistically significant at the 0.1% level. In column (3) of Table 1, we include the policy adoption density measure [cf. 4] and historical rainfall patterns, which do not substantially affect the estimate on residential movement.

Columns (4) and (5) of Table 1 estimate the impact of walking mobility using the Apple data. Including our standard battery of controls, column (4) estimates a statistically significant (at 0.1% level) negative relationship between lagged walking mobility and policy stringency. This estimated negative effect is consistent with the previous results on staying put. Geographic mobility out of residential areas (by walking) leads to less stringent COVID-19 policy responses. According to our calculations, one standard deviation increase in walking movement is associated with a 0.11 standard deviation decrease in the policy stringency index. In column (5), we have also included the policy adoption density and historical rainfall controls.

### Two-Stage Least Squares Results

In this subsection we implement our instrumental variable strategy, replicating columns 2–5 from Table 1. In Table 2 we show in Panel A the coefficient on our excluded rainfall instrument in the first stage regression, while Panel B shows the second stage regression output for the variables of interest. To conserve space, we suppress the estimates for the control variables. As expected, more rainfall is associated with people staying put more at residential locations and less walking mobility. The first stage impact of rainfall on our preferred mobility variable (residential) is highly statistically significant (usually at the 0.1% level) and the relevant first stage diagnostic, the K-P F-statistic, is above its threshold value of ten. Since the Cragg-Donald (C-D) F-statistic assumes homoskedastic errors, the Kleibergen-Paap (K-P) F-statistic, which is valid under non-i.i.d errors, is more reliable for our data. Thus the relevance criteria for a valid instrument is satisfied.

**TABLE 2:** Two-Stage Least Squares regressions—Stringency index (Worldwide, 2020).

	(1)	(2)	(3)	(4)
Dependent variable: Mobility (7-day moving averages)				
Panel A: First-Stage				
	Residential	Residential	Walking	Walking
Rainfall (7-day moving averages)	1.035*** (0.277)	0.961** (0.300)	−8.197* (3.833)	−7.472 (4.738)
Dependent variable: Stringency index				
Panel B: Second-Stage				
Residential (7-day moving averages)	2.288*** (0.475)	1.873** (0.571)		
Walking (7-day moving averages)			−0.391* (0.159)	−0.396+ (0.236)
First-stage C-D F-stat	23.178	18.306	6.115	4.718
First-stage K-P F-stat	13.935	10.230	4.575	2.488
Country-days	192	191	216	215
N	11,009	10,920	4,574	4,562
Complete controls	✓	✓	✓	✓
Region fixed effects	✓	✓	✓	✓
Day fixed effects	✓	✓	✓	✓

Notes: All specifications include Driscoll-Kraay (DK) standard errors (in parenthesis). The full table with the coefficients of the control variables is reported in the Supplementary Appendix. ^+^ *p* < 0.1, *p < 0.05, **p < 0.01, ***p < 0.001.

The second stage estimations, shown in Panel B of Table 2, are consistent with the results from the OLS estimations. Staying put at residential locations is positively associate with policy stringency and walking mobility is negatively associated. In column 2, one standard deviation increase in residential movement is associated with 0.54 standard deviation increase in the stringency index. This increase in the magnitude is consistent with the supposition that OLS was under-estimating the impact due to a reverse causality bias. For example, if households anticipate an increase in policy stringency tomorrow, they may increase their mobility levels today (staying home less and walking more), so the reverse causal mechanism would predict a positive relation which would deflate the negative coefficient if not corrected for.

The main identifying assumption is that the instrument satisfies the “exclusion restriction,” which requires that the instrument (rainfall) affects the dependent variable (policy stringency) only through its impact on the potentially endogenous variable (mobility). One possible violation of the exclusion restriction would be if rainy weather leads to an uptick in colds and flus, which may put a strain on public health care systems and make public officials more sensitive to upticks in coronavirus cases (for any level of mobility). We believe that our control for hospital beds per 1,000 people effectively deals with this channel. A second possible violation may be that extreme rainfall conditions (droughts and floods, eg) lead directly to some governmental restrictions on public life (school and public transport closures, eg) that may get lumped together with other COVID-19 policy responses. On this possibility, we note that closures of public services are only a fraction of the overall index of policy stringency that we use.

### Investigation of “Channels”

At first glance, the estimated negative relationship between geographic mobility and policy stringency may seem counter-intuitive. After all, more mobile societies are likely to transport and transmit the virus at a higher rate than less mobile societies, so the public health benefits of stringent policy responses should be higher [13], which would imply a positive association between mobility and policy stringency. On the other hand, it may be more economically and politically costly for policymakers to impose stringent regulations on a more geographically mobile society [14, 15], which would imply a negative association between mobility and policy stringency. In considering these costs and benefits of policy stringency as a function of mobility, our estimations in Tables 1, 2 imply that on net, higher mobility seems to impact the cost side of policy stringency more than the benefit side.

In order to investigate the cost channels that may explain the negative relation between geographic mobility and stringency of policy responses, we have analyzed some more focused categories of mobility. Specifically, we look at movements related to commerce and those related to civil society. In Table 3 we analyze Google movement data that relates to “retail and recreation,” “grocery and pharmacy,” “parks and recreation,” as well as “protests” and “riots” from COVID-19 Disorder Tracker. The TSLS results indicate that the commercial and leisure movement variables are quite significantly negatively related to policy stringency. The estimates on the civil society movements are weaker. The estimated effect of protest movement is statistically insignificant and negative (though we note that the lower strength of the rainfall instrument for this kind of movement may be driving the insignificant result), while the estimated negative effect of riot is borderline statistical significant (*p* < 0.1) and parks is statistically significant and negative (*p* < 0.05). In column (1), one standard deviation in retail and recreation mobility decreases the stringency index by 0.26 standard deviations. In column (2), one standard deviation in grocery and pharmacy mobility decreases the stringency index by 0.26 standard deviations. In column (3), one standard deviation increase in workplaces mobility decreases the stringency index by 0.36 standard deviations.

**TABLE 3:** Two-Stage Least Squares regressions—Business vs. civil society (Worldwide, 2020).

	(1)	(2)	(3)	(4)	(5)	(6)
Dependent variable: Stringency index; Instrument: Rainfall (7-day moving averages)						
Retail and recreation (7-day moving avgs)	−0.528** (0.161)					
Grocery and Pharmacy (7-day moving avgs)		−0.443** (0.154)				
Workplaces (7-day moving averages)			−0.596* (0.238)			
Protests (7-day moving averages)				−106.250 (379.767)		
Riots (7-day moving averages)					−36.613+ (21.473)	
Parks (7-day moving averages)						−0.288* (0.124)
First-stage C-D F-stat	53.011	77.972	41.567	0.217	22.828	32.336
First-stage K-P F-stat	23.216	37.279	17.086	0.076	4.729	14.661
Country-days	191	191	191	236	236	191
N	10,860	10,860	10,918	12,210	12,210	10,803
Complete controls	✓	✓	✓	✓	✓	✓
Region fixed effects	✓	✓	✓	✓	✓	✓
Day fixed effects	✓	✓	✓	✓	✓	✓

Notes: All specifications include Driscoll-Kraay (DK) standard errors (in parenthesis). The full table with the coefficients of the control variables is reported in the Supplementary Appendix. ^+^ *p* < 0.1, *p < 0.05, **p < 0.01, ***p < 0.001.

The impact of movement related to commercial activity is a robust explanatory factor of policy stringency, while civil society movements do not have such a clear impact. In Supplementary Table SA.13, we show that a variety of civil unrest measures from conflicts that are not related to COVID-19 do not correlate with policy stringency. Policy-makers’ decision-making should not respond to these “placebo” treatments (unrelated to the policy issue) and that is indeed what we find.

**FIGURE 1:**
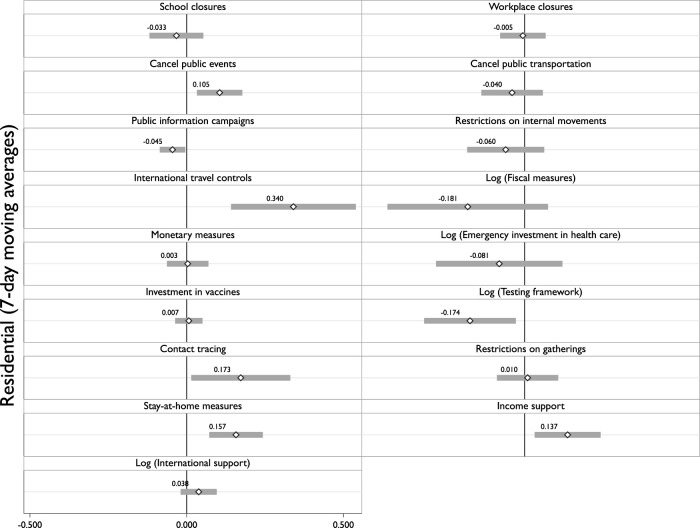
Disaggregated policy responses. All Two-Stage Least Squares regression models include the same controls as column 3 from Table 1. Coefficients are represented by open dots and their respective numbers. Bars are 95% confidence intervals, calculated with Driscoll-Kraay standard errors (Worldwide, 2020).

Furthermore, we have taken a look at finer grained policy response measures. Those policy areas are school closures, workplace closures, canceling public events, closing public transport, public information campaigns, restrictions on internal movement, international travel controls, fiscal measures, monetary measures, emergency investment in health care, investment in vaccines, testing frameworks, contact tracing, restrictions on gatherings, stay-at-home measures, income support, and international support. We now examine how the individual policy components of the composite index respond to the instrumented variation in geographic mobility. In Figure 1, we show statistically significantly positive associations between instrumented residential movement and policies such as contact tracing, canceling public events, stay-at-home orders, regulating international travel control, and income supports. On the other hand, we also document statistically significant negative associations between instrumented movement and policies that regulate public information campaigns and testing frameworks. All statistically significant variables are ordinal variables, registering progressively higher levels of intensity of policy responses. Although our results are heterogeneous, statistically significant positive variables tend to reduce mobility, increasing business costs. In contrast, significant negative variables tend to improve prevention, not substantially affecting commercial activities. Therefore, the disaggregated analysis follows a pattern consistent with our previous results.

### Further Robustness Analysis

First, readers may be concerned that the results are being driven by specific countries. We have performed “leave-one-out” checks that drop the lowest and highest mobility countries for both the OLS and TSLS models, which are graphically summarized in Supplementary Figure SA.2. Supplementary Tables SA.4–SA.6 report additional results concerning the rainfall IV, namely the first-stage estimates, the reduced form estimates, and alternative constructions of the instrument (the continuous variable and binary variables with alternative thresholds). Concerning our battery of controls, we have also considered some alternatives. For instance, we have also reproduced Tables 1, 2 with the same controls found in [4] in Supplementary Tables SA.7, SA.8. Some readers may find our use of a seven-day lagged moving average to be arbitrary, so Supplementary Table SA.9 reproduces the main TSLS results using alternative lag structures, namely moving averages of 5, 10, and 14 days, respectively.

We have dropped the extreme rainfall observations (Supplementary Table SA.10), dropped the largest countries in terms of their population (Supplementary Table SA.11) and geography (Supplementary Table SA.12). We also have employed quadratic effects of the IV’s (Supplementary Table SA.14). Concerning the instrument, we have considered alternative operationalizations, such as using the raw data, an inverse hyperbolic sine transformation, and alternative thresholds for the binary variable (Supplementary Tables SA.6, SA.15). The instruments are often much stronger in these alternative operationalizations, with first-stage K-P F-statistics greater than 47, and coefficients are also remarkably stable, ranging from 2.1 to 2.6. As our baseline results in column 3 from Table 2 is 2.0, we present a slightly conservative estimate in the main paper. Supplementary Figure SA.3 explores the sensitivity of IV estimates to potential violations of the exclusion restriction, which is described in Supplementary Section SA.2. We have also considered alternative climate variables, such as maximum wind speed, volatility of wind speed and visibility conditions in Supplementary Table SA.16. To deal with spill-over effects from our rainy instrument, columns 2 and 3 from Supplementary Table SA.20 add countries’ latitude and longitude as controls. To avoid ecological inferences, column 1 from Supplementary Table SA.20 estimates the paper’s baseline model by adding a control for federal political systems, which present more within-country variation. We additionally check the robustness of the results when controlling for subnational variation of policies. In Supplementary Figure SA.4, we show how nationally-measured stringency of disaggregated policy responses are affected by mobility controlling for whether policies are targeted to specific geographical regions.

## Discussion

Previous research (see Supplementary Table SA.21) shows that measures to reduce mobility reduce COVID-19 cases, but these policies are motivated more by political and economic incentives than by strict public health considerations. Specifically, reduction in connectivity is more vital for municipalities with a low average income in Italy [16]. However, wealthy areas went from most mobile before the pandemic to least mobile in France, Italy, and the U.K. [17], and areas showing higher resilience to mobility disruptions are those where GDP per capita is higher in the U.S [18]. This paper has established that governments’ policy responses to the COVID-19 pandemic are to some extent determined by how mobile their societies are. While shutting down movement should have the largest public health benefit in highly mobile societies, it may also have the largest political and economic costs. Our analysis demonstrates that geographic mobility may have an important role in explaining why some countries have pursued more stringent policy responses than others.
