# Supplementary material for: Mobility and Policy Responses During the COVID-19 Pandemic in 2020
Source: Int J Public Health. 2022 Aug 5;67:1604663. doi: 10.3389/ijph.2022.1604663 (PMC9389530; doi:10.3389/ijph.2022.1604663)
Supplement: Supplementary file 1 [file Datasheet1.PDF]

# Mobility and policy responses during the COVID-19 pandemic in 2020

## Online Appendix

May 29, 2022

**Abstract**

***Objective.*** This paper quantitatively explores determinants of governments' non-pharmaceutical policy responses to the COVID-19 pandemic. Our focus is on the extent to which geographic mobility affected the stringency of governmental policy responses. ***Methods.*** Using cross-country, daily frequency data on geographic mobility and COVID-19 policy stringency during 2020, we investigate some of the determinants of policy responses to COVID-19. In order to causally identify the effect of geographic mobility on policy stringency, we pursue an instrumental variable strategy that exploits climate data to identify arguably exogenous variation in geographic mobility. ***Results.*** We find that societies that are more geographically mobile have governmental policy responses that are less stringent. Examining disaggregated mobility data, we show that the negative relation between geographic mobility and policy stringency is the stronger for commercially-oriented movements than for geographic movements that relate to civil society. ***Conclusion***. The results suggest that policy-makers are more willing to trade-off public health for economic concerns relative to other civil concerns.

**Keywords:** COVID-19, Geographic mobility, Policy responses, High-frequency panel data, Instrumental variable regressions

# A Online Appendix – Not for Print Publication

Table A.1: Non-advanced CIA countries and their respective regions.

| | |
|---|---|
| Eastern Europe and post Soviet Union | Albania, Armenia, Azerbaijan, Belarus, Bosnia and Herzegovina, Bulgaria, Croatia, Georgia, Hungary, Kazakhstan, Kyrgyzstan, Moldova, Montenegro, North Macedonia, Poland, Romania, Russia, Serbia, Tajikistan, Turkmenistan, Ukraine, Uzbekistan |
| Latin America | Argentina, Bolivia, Brazil, Chile, Colombia, Costa Rica, Cuba, Dominican Republic, Ecuador, El Salvador, Falkland Islands, French Guiana, Guatemala, Haiti, Honduras, Mexico, Nicaragua, Panama, Paraguay, Peru, South Georgia and South Sandwich Islands, Turks and Caicos Islands, Uruguay, Venezuela |
| North Africa and the Middle East | Algeria, Bahrain, Egypt, Iran, Iraq, Jordan, Kuwait, Lebanon, Libya, Morocco, Oman, Palestinian Territories, Qatar, Saudi Arabia, Syria, Tunisia, Turkey, United Arab Emirates, Western Sahara, Yemen |
| Sub-Saharan Africa | Angola, Benin, Botswana, Burkina Faso, Burundi, Cameroon, Cape Verde, Central African Republic, Chad, Comoros, Congo - Brazzaville, Congo - Kinshasa, Côte d'Ivoire, Djibouti, Equatorial Guinea, Eritrea, Eswatini, Ethiopia, French Southern Territories, Gabon, Gambia, Ghana, Guinea, Guinea-Bissau, Kenya, Lesotho, Liberia, Madagascar, Malawi, Mali, Mauritania, Mauritius, Mayotte, Mozambique, Namibia, Niger, Nigeria, Rwanda, Réunion, Senegal, Seychelles, Sierra Leone, Somalia, South Africa, South Sudan, St. Helena, Sudan, São Tomé and Príncipe, Tanzania, Togo, Uganda, Zambia, Zimbabwe |
| Western Europe and North America | Gibraltar, Greenland, Isle of Man, St. Pierre and Miquelon, Svalbard and Jan Mayen |
| East Asia | China, Mongolia, North Korea |
| South-East Asia | Brunei, Cambodia, Christmas Island, Cocos (Keeling) Islands, Indonesia, Laos, Malaysia, Myanmar (Burma), Philippines, Thailand, Timor-Leste, Vietnam |
| South Asia | Afghanistan, Bangladesh, Bhutan, British Indian Ocean Territory, India, Maldives, Nepal, Pakistan, Sri Lanka |
| The Pacific | American Samoa, Cook Islands, Fiji, French Polynesia, Guam, Kiribati, Marshall Islands, Micronesia (Federated States of), New Caledonia, Niue, Norfolk Island, Northern Mariana Islands, Palau, Papua New Guinea, Pitcairn Islands, Samoa, Solomon Islands, Tokelau, Tonga, Tuvalu, United States Minor Outlying Islands (the), Vanuatu, Wallis and Futuna |
| The Caribbean | Anguilla, Antigua and Barbuda, Aruba, Bahamas, Barbados, Belize, British Virgin Islands, Cayman Islands, Curaçao, Dominica, Grenada, Guadeloupe, Guyana, Jamaica, Martinique, Montserrat, Sint Maarten, St. Kitts and Nevis, St. Lucia, St. Vincent and Grenadines, Suriname, Trinidad and Tobago, U.S. Virgin Islands |

Table A.2: Summary statistics (main paper)

| Variable | Obs | Mean | Std. Dev. | Min | Max |
|---|---|---|---|---|---|
| Stringency index | 34278 | 48.148 | 34.044 | 0 | 100 |
| Residential | 19003 | 11.818 | 10.299 | -35 | 52 |
| Residential (7-day moving averages) | 17783 | 12.065 | 9.806 | -13.375 | 42.75 |
| Walking | 6440 | 82.291 | 61.555 | 6.96 | 888.44 |
| Walking (7-day moving averages) | 6048 | 82.557 | 61.558 | 9.328 | 793.898 |
| Retail and Recreation | 19185 | -29.044 | 26.541 | -98 | 54 |
| Retail and recreation (7-day moving avgs) | 18388 | -29.79 | 25.304 | -93 | 27.375 |
| Grocery and Pharmacy | 19168 | -14.369 | 22.42 | -98 | 162 |
| Grocery and Pharmacy (7-day moving avgs) | 18267 | -14.63 | 20.129 | -85.75 | 62.625 |
| Workplaces | 19502 | -23.217 | 22.819 | -93 | 43 |
| Workplaces (7-day moving averages) | 18661 | -23.829 | 20.549 | -83.75 | 24.5 |
| Protests | 32208 | .293 | 1.724 | 0 | 122 |
| Protests (7-day moving averages) | 31284 | .297 | 1.198 | 0 | 31.875 |
| Riots | 32208 | .051 | .411 | 0 | 22 |
| Riots (7-day moving averages) | 31284 | .053 | .31 | 0 | 8.375 |
| Parks | 19051 | -16.824 | 39.299 | -100 | 484 |
| Parks (7-day moving averages) | 18151 | -17.483 | 37.87 | -92.875 | 424.125 |
| Rainfall | 43771 | .227 | .419 | 0 | 1 |
| Rainfall (7-day moving averages) | 39450 | .238 | .278 | 0 | 1 |
| Cancel public events | 34413 | 1.198 | .946 | 0 | 2 |
| Close public transport | 34540 | .601 | .8 | 0 | 2 |
| Public information campaigns | 33944 | 1.506 | .803 | 0 | 2 |
| Restrict internal movement | 34491 | .903 | .942 | 0 | 2 |
| Inttravel controls | 34429 | 2.472 | 1.62 | 0 | 4 |
| Log (Fiscal measures) | 32698 | 1.10e+08 | 7.51e+09 | 0 | 1.19e+12 |
| Monetary measures | 33026 | .696 | .831 | 0 | 2 |
| Log (Emergency invest health care) | 32654 | 2863792 | 1.55e+08 | 0 | 1.94e+10 |
| Log (Invest vaccines) | 32663 | 189006.7 | 2.09e+07 | 0 | 3.44e+09 |
| Testing framework | 33582 | 1.195 | .963 | 0 | 3 |
| Contact tracing | 33572 | 1.027 | .855 | 0 | 2 |
| Restrictions on gatherings | 34379 | 1.961 | 1.734 | 0 | 4 |
| Stay-at-home measures | 34379 | .916 | 1.009 | 0 | 3 |
| Income support | 33464 | .502 | .673 | 0 | 2 |
| Log (International support) | 32716 | .016 | .517 | 0 | 24.25 |
| Level of Democracy (Freedom House/Imputed Polity) | 37293 | 5.917 | 2.977 | 0 | 10 |
| ICRG Indicator of Quality of Government | 25376 | .436 | .128 | .083 | .75 |
| Log (Confirmed cases) | 25784 | 6.478 | 3.414 | 0 | 15.167 |
| Log (Real GDP per capita) | 31196 | 8.844 | 1.08 | 6.428 | 11.848 |
| Population density (people per sq. km of land area) | 36561 | 145.334 | 239.685 | 1.967 | 1832.636 |
| Population ages 65 and above (% of total population) | 35832 | 6.161 | 4.212 | .987 | 20.429 |
| Trade (% of GDP) | 34891 | 78.566 | 33.812 | 20.723 | 184.686 |
| Hospital beds (per 1,000 people) | 22806 | 2.509 | 2.066 | .1 | 11 |
| SARS | 48031 | 27.641 | 378.705 | 0 | 5327 |
| Log (Airports) | 45063 | 3.33 | 1.69 | .693 | 8.317 |
| Adoption density | 34278 | 58.978 | 49.46 | 0 | 226.28 |
| Historical rainfall | 44088 | .1 | .293 | 0 | 8.43 |

Table A.3: Summary statistics (appendix)

| Variable | Obs | Mean | Std. Dev. | Min | Max |
|---|---|---|---|---|---|
| School closing | 34440 | 1.738 | 1.395 | 0 | 3 |
| Workplace closing | 34338 | 1.19 | 1.15 | 0 | 3 |
| Residential (5-day moving averages) | 18045 | 11.974 | 9.854 | -17.333 | 44 |
| Residential (10-day moving averages) | 17477 | 12.194 | 9.732 | -11.182 | 42.909 |
| Residential (14-day moving averages) | 17069 | 12.365 | 9.619 | -9.867 | 42.2 |
| PRCP | 43771 | .103 | .302 | 0 | 15.03 |
| Precipitation (hundreth of inches) (7-day moving averages) | 39450 | .107 | .174 | 0 | 2.575 |
| PRCP (hundreth of inches, IHS) | 43771 | .092 | .215 | 0 | 3.404 |
| Precipitation (hundreth of inches, IHS) (7-day moving averages) | 39450 | .096 | .135 | 0 | 1.487 |
| Rainfall (7-day moving averages) squared | 39450 | .134 | .23 | 0 | 1 |
| PRCP (hundreth of inches) squared | 39450 | .093 | .129 | 0 | 1.274 |
| PRCP (hundreth of inches, IHS) squared | 39450 | .086 | .109 | 0 | .911 |
| Rapid fluct. of wind speed (7-day moving averages) | 6980 | 23.413 | 4.991 | 12.714 | 48.525 |
| Max. wind speed (7-day moving averages) | 42714 | 11.918 | 3.906 | 2.484 | 35.388 |
| Visibility (7-day moving averages) | 41529 | 8.428 | 3.63 | 1.322 | 27.992 |
| Electoral democracy index | 33636 | .464 | .22 | .022 | .893 |
| Death rate (per 100,000) | 25784 | 10.653 | 60.327 | 0 | 1208.28 |
| Tax revenue (% of GDP) | 25863 | 15.551 | 6.248 | 0 | 31.674 |
| GINI index (World Bank estimate) | 20496 | 40.207 | 7.725 | 25 | 63 |
| Urban population (% of total population) | 37049 | 52.718 | 21.613 | 12.388 | 100 |

## A.1 Control variables

In tables A.17 – A.19 at the end of the online appendix, we provide sources and descriptions for all the variables used in the study. As mentioned in the main text, we have followed previous literature on COVID-19 policy responses [7, 26] in our selection of control variables. In this section we briefly describe the rationale for inclusion of these variables.

The controls that we include in the analysis help to prevent our results from being biased due to the omission of relevant variables. Omitted variable bias is a concern if an omitted variable is correlated with the outcome variable (COVID-19 policy stringency) and with the explanatory variable of interest (geographic mobility). As noted in the main text, some of the variables are also available at a daily frequency (noted below parenthetically). For control that are only available at a yearly frequency, we repeat the value daily.

First, we control for measures of COVID-19 incidence in the population, as the health of the population may be correlated with how much people move around and also the stringency of public policy responses. This measure is the log of COVID-19 cases (daily variation).

Second, we control for measures of the susceptibility of population to the transmission of COVID-19. These controls include the percentage of the population over the age of 65 (older populations are more likely to die from COVID-19 infection), the density of the population (the virus is more likely to be spread in dense populations), and the log of the number of airports in the country (the virus is more likely to spread geographically with more travelling), and the openness of the economy to the rest of the world (more international economic exchanges are more likely to spread the virus).

Third, we control for measures of state capacity, in general and also with regards to public health: the level of democracy and an indicator of the government's capacity to maintain law and order (which may affect both policy responses and mobility), a dummy for experiencing SARS that takes value one if there were more than 100 confirmed cases of SARS in the country in 2002 – 2003 (policy-makers may be more responsive and the population more cautious if there was experience with SARS), and the number of hospital beds per 1,000 people (policy-makers and the population may be less cautious if the health care system has strong capacity to care for serious cases).

Finally, we include two very broad controls that should be correlated with many elements of public health policy and the mobility of the population: the level of economic development, proxied by the national economy's real gross domestic product per capita, and the adoption density of the stringency of COVID-19 policy responses

in the country's geographical region (daily frequency). As for the level of economic development, more developed economies will have more commercially-oriented geographical mobility and may have systematically different public health capacities and policy approaches. The policy adoption density is a powerful time-varying regional control as policies and social cultural norms of mobility are likely to have been geo-spatially correlated during the early stages of the pandemic.

## Table A.4: Two-Stage Least Squares regressions — Stringency index

| ***Dependent variable: Mobility (7-day moving averages)*** | | | | |
|---|---|---|---|---|
| *Panel A: First-Stage* | (1) Residential | (2) Residential | (3) Walking | (4) Walking |
| Rainfall (7-day moving averages) | 1.035*** | 0.961** | -8.197* | -7.472 |
| | (0.277) | (0.300) | (3.833) | (4.738) |
| Level of Democracy (Freedom House/Imputed Polity) | 0.828*** | 0.813*** | -0.231 | -0.162 |
| | (0.040) | (0.041) | (0.441) | (0.456) |
| ICRG Indicator of Quality of Government | 6.316*** | 6.384*** | 26.330* | 20.779 |
| | (0.508) | (0.508) | (13.075) | (12.773) |
| Log (Confirmed cases) | 0.946*** | 0.938*** | -8.654*** | -8.640*** |
| | (0.056) | (0.057) | (0.960) | (0.979) |
| Log (Real GDP per capita) | 1.941*** | 1.961*** | 21.807*** | 22.766*** |
| | (0.121) | (0.121) | (2.417) | (2.534) |
| Population density (people per sq. km of land area) | -0.001*** | -0.001*** | -0.056*** | -0.052*** |
| | (0.000) | (0.000) | (0.005) | (0.007) |
| Population ages 65 and above (% of total population) | -0.683*** | -0.687*** | 5.563*** | 5.708*** |
| | (0.032) | (0.032) | (1.320) | (1.343) |
| Trade (% of GDP) | 0.022*** | 0.021*** | -0.092** | -0.079** |
| | (0.002) | (0.002) | (0.029) | (0.030) |
| Hospital beds (per 1,000 people) | 0.356*** | 0.332*** | -17.931*** | -18.414*** |
| | (0.052) | (0.051) | (3.244) | (3.270) |
| SARS | -0.054*** | -0.054*** | 1.715*** | 1.717*** |
| | (0.012) | (0.012) | (0.168) | (0.168) |
| Log (Airports) | -0.671*** | -0.672*** | 24.197*** | 24.615*** |
| | (0.062) | (0.061) | (3.489) | (3.499) |
| Adoption density | | 0.085*** | | -0.766*** |
| | | (0.009) | | (0.121) |
| Historical rainfall | | 0.675 | | -8.127 |
| | | (0.453) | | (11.772) |
| ***Dependent variable: Stringency index*** | | | | |
| *Panel B: Second-Stage* | (1) | (2) | (3) | (4) |
| Residential (7-day moving averages) | 2.288*** | 1.873** | | |
| | (0.475) | (0.571) | | |
| Walking (7-day moving averages) | | | -0.391* | -0.396+ |
| | | | (0.159) | (0.236) |
| Level of Democracy (Freedom House/Imputed Polity) | 0.968* | 1.359** | -3.351*** | -3.359*** |
| | (0.398) | (0.473) | (0.325) | (0.332) |
| ICRG Indicator of Quality of Government | -47.977*** | -45.551*** | 65.183*** | 64.585*** |
| | (3.582) | (4.069) | (8.819) | (9.417) |
| Log (Confirmed cases) | -0.263 | 0.135 | 0.963 | 0.912 |
| | (0.510) | (0.598) | (1.456) | (2.132) |
| Log (Real GDP per capita) | 0.777 | 1.644 | -6.675+ | -6.444 |
| | (0.872) | (1.081) | (3.410) | (5.353) |
| Population density (people per sq. km of land area) | 0.006*** | 0.005*** | 0.011 | 0.011 |
| | (0.001) | (0.001) | (0.013) | (0.015) |
| Population ages 65 and above (% of total population) | -0.803** | -1.113** | -0.048 | 0.007 |
| | (0.290) | (0.362) | (1.062) | (1.504) |
| Trade (% of GDP) | -0.082*** | -0.070*** | -0.115*** | -0.113*** |
| | (0.012) | (0.013) | (0.018) | (0.022) |
| Hospital beds (per 1,000 people) | -1.530*** | -1.402*** | -3.390 | -3.560 |
| | (0.194) | (0.203) | (3.257) | (4.683) |
| SARS | 0.609*** | 0.585*** | 0.333 | 0.338 |
| | (0.030) | (0.033) | (0.299) | (0.426) |
| Log (Airports) | 1.701*** | 1.455** | 4.222 | 4.426 |
| | (0.385) | (0.446) | (4.267) | (6.164) |
| Adoption density | | -0.007 | | -0.111 |
| | | (0.057) | | (0.196) |
| Historical rainfall | | 1.681+ | | -0.714 |
| | | (1.002) | | (6.013) |
| First-stage C-D F-stat | 23.178 | 18.306 | 6.115 | 4.718 |
| First-stage K-P F-stat | 13.935 | 10.230 | 4.575 | 2.488 |
| Country-days | 192 | 191 | 216 | 215 |
| N | 11009 | 10920 | 4574 | 4562 |
| Region fixed effects | ✓ | ✓ | ✓ | ✓ |
| Day fixed effects | ✓ | ✓ | ✓ | ✓ |

*Notes*: All specifications include Driscoll-Kraay (DK) standard errors (in parenthesis). DK non-parametric standard errors are heteroskedasticity robust to cross-country and day dependences and autocorrelated consistent (up to three-day lags). $+p < .1, *p < .05, **p < .01, ***p < .001$.

Table A.5: Reduced form — Stringency index

| | (1) | (2) | (3) |
|---|---|---|---|
| ***Dependent variable: Stringency index*** | | | |
| Rainfall (7-day moving averages) | 10.522*** | 3.341*** | 2.456*** |
| | (1.725) | (0.720) | (0.744) |
| Level of Democracy (Freedom House/Imputed Polity) | | 0.611*** | 0.686*** |
| | | (0.137) | (0.135) |
| ICRG Indicator of Quality of Government | | -8.025*** | -9.196*** |
| | | (1.655) | (1.798) |
| Log (Confirmed cases) | | 2.219*** | 2.058*** |
| | | (0.251) | (0.237) |
| Log (Real GDP per capita) | | 2.024*** | 2.340*** |
| | | (0.354) | (0.341) |
| Population density (people per sq. km of land area) | | 0.003*** | 0.003*** |
| | | (0.001) | (0.001) |
| Population ages 65 and above (% of total population) | | -1.646*** | -1.691*** |
| | | (0.078) | (0.086) |
| Trade (% of GDP) | | -0.048*** | -0.047*** |
| | | (0.005) | (0.005) |
| Hospital beds (per 1,000 people) | | -0.790*** | -0.758*** |
| | | (0.122) | (0.133) |
| SARS | | 0.003*** | 0.003*** |
| | | (0.000) | (0.000) |
| Log (Airports) | | -0.992*** | -0.971*** |
| | | (0.190) | (0.189) |
| Adoption density | | | 0.186*** |
| | | | (0.020) |
| Historical rainfall | | | 1.740** |
| | | | (0.601) |
| R-squared | 0.008 | 0.172 | 0.189 |
| Country-days | 237 | 237 | 236 |
| N | 27910 | 13100 | 12943 |
| Region fixed effects | | ✓ | ✓ |
| Day fixed effects | | ✓ | ✓ |

*Notes*: All specifications include Driscoll-Kraay (DK) standard errors (in parenthesis). DK non-parametric standard errors are heteroskedasticity robust to cross-country and day dependences and autocorrelated consistent (up to three-day lags). $+p < .1, *p < .05, **p < .01, ***p < .001$.

Table A.6: Two-Stage Least Squares regressions — Stringency index — Robustness check

| | Instruments: Rainfall measures (7-day moving averages) | | | | |
|---|---|---|---|---|---|
| | (1) | (2) | (3) | (4) | (5) |
| | PRCP (hundreth of inches) | PRCP (hundreth of inches, IHS) | Rainfall (> 0.2 inches) | Rainfall (> 0.3 inches) | Rainfall (> 0.35 inches) |
| ***Dependent variable: Stringency index*** | | | | | |
| Residential (7-day moving averages) | 2.359*** | 2.430*** | 1.772*** | 2.202*** | 2.137*** |
| | (0.276) | (0.311) | (0.422) | (0.316) | (0.386) |
| Level of Democracy (Freedom House/Imputed Polity) | 0.964*** | 0.906*** | 1.441*** | 1.092*** | 1.145*** |
| | (0.242) | (0.266) | (0.349) | (0.280) | (0.343) |
| ICRG Indicator of Quality of Government | -48.771*** | -49.244*** | -44.878*** | -47.730*** | -47.299*** |
| | (2.492) | (2.728) | (3.405) | (2.894) | (3.159) |
| Log (Confirmed cases) | -0.328 | -0.395 | 0.231 | -0.178 | -0.116 |
| | (0.281) | (0.320) | (0.412) | (0.335) | (0.364) |
| Log (Real GDP per capita) | 0.714 | 0.578 | 1.839* | 1.015+ | 1.140+ |
| | (0.504) | (0.551) | (0.784) | (0.549) | (0.664) |
| Population density (people per sq. km of land area) | 0.006*** | 0.006*** | 0.005*** | 0.006*** | 0.006*** |
| | (0.001) | (0.001) | (0.001) | (0.001) | (0.001) |
| Population ages 65 and above (% of total population) | -0.784*** | -0.736*** | -1.181*** | -0.891*** | -0.935*** |
| | (0.201) | (0.217) | (0.287) | (0.225) | (0.276) |
| Trade (% of GDP) | -0.081*** | -0.082*** | -0.068*** | -0.077*** | -0.076*** |
| | (0.009) | (0.010) | (0.010) | (0.009) | (0.011) |
| Hospital beds (per 1,000 people) | -1.556*** | -1.578*** | -1.370*** | -1.506*** | -1.486*** |
| | (0.210) | (0.212) | (0.212) | (0.214) | (0.230) |
| SARS | 0.613*** | 0.617*** | 0.579*** | 0.604*** | 0.600*** |
| | (0.030) | (0.031) | (0.034) | (0.033) | (0.037) |
| Log (Airports) | 1.784*** | 1.833*** | 1.386*** | 1.678*** | 1.633*** |
| | (0.242) | (0.266) | (0.330) | (0.266) | (0.300) |
| Adoption density | -0.048 | -0.054 | 0.002 | -0.035 | -0.029 |
| | (0.033) | (0.036) | (0.043) | (0.032) | (0.037) |
| Historical rainfall | 1.146 | 1.067 | 1.793* | 1.319 | 1.391 |
| | (0.867) | (0.912) | (0.832) | (0.876) | (0.885) |
| First-stage C-D F-stat | 68.709 | 57.494 | 31.522 | 48.894 | 51.216 |
| First-stage K-P F-stat | 39.007 | 30.559 | 15.589 | 21.221 | 21.534 |
| Country-days | 191 | 191 | 191 | 191 | 191 |
| N | 10920 | 10920 | 10920 | 10920 | 10920 |
| Region fixed effects | ✓ | ✓ | ✓ | ✓ | ✓ |
| Day fixed effects | ✓ | ✓ | ✓ | ✓ | ✓ |

*Notes*:All specifications include Driscoll-Kraay (DK) standard errors (in parenthesis). DK non-parametric standard errors are heteroskedasticity robust to cross-country and day dependences and autocorrelated consistent (up to three-day lags). $+p < .1, *p < .05, **p < .01, ***p < .001$.

Table A.7: OLS regressions — Stringency index

| | (1) | (2) | (3) | (4) | (5) |
|---|---|---|---|---|---|
| ***Dependent variable: Stringency index*** | | | | | |
| Residential (7-day moving averages) | 1.883*** | 1.877*** | 1.814*** | 1.877*** | 1.895*** |
| | (0.035) | (0.036) | (0.036) | (0.036) | (0.036) |
| Log (Real GDP per capita) | 0.521 | 0.568 | -0.485 | -0.540 | -0.546 |
| | (0.398) | (0.396) | (0.362) | (0.401) | (0.402) |
| Tax revenue (% of GDP) | -0.901*** | -0.935*** | -0.962*** | -0.940*** | -0.967*** |
| | (0.070) | (0.077) | (0.074) | (0.067) | (0.064) |
| GINI index (World Bank estimate) | -0.039 | 0.030 | 0.042 | -0.027 | 0.015 |
| | (0.046) | (0.048) | (0.047) | (0.045) | (0.048) |
| Hospital beds (per 1,000 people) | -2.762*** | -2.762*** | -2.196*** | -2.320*** | -2.182*** |
| | (0.197) | (0.195) | (0.208) | (0.200) | (0.211) |
| Population ages 65 and above (% of total population) | -0.332* | -0.330* | -0.627*** | -0.530*** | -0.484*** |
| | (0.153) | (0.151) | (0.144) | (0.143) | (0.142) |
| Urban population (% of total population) | -0.010 | 0.002 | 0.007 | -0.001 | -0.027 |
| | (0.016) | (0.016) | (0.016) | (0.016) | (0.018) |
| Log (Population density) | -0.149 | -0.219 | -0.244 | -0.231 | -0.088 |
| | (0.175) | (0.177) | (0.174) | (0.178) | (0.169) |
| Death rate (per 100,000) | 0.018*** | | | 0.017*** | 0.016*** |
| | (0.002) | | | (0.002) | (0.002) |
| Adoption density | | -0.070** | | -0.078** | -0.001 |
| | | (0.021) | | (0.024) | (0.022) |
| Electoral democracy index | | | 10.814*** | 8.358*** | 24.593*** |
| | | | (1.095) | (1.114) | (3.542) |
| Adoption density × Electoral democracy index | | | | | -0.165*** |
| | | | | | (0.034) |
| R-squared | 0.428 | 0.419 | 0.423 | 0.433 | 0.436 |
| Country-days | 192 | 192 | 192 | 192 | 192 |
| N | 8088 | 8329 | 8329 | 8088 | 8088 |
| Region fixed effects | ✓ | ✓ | ✓ | ✓ | ✓ |
| Day fixed effects | ✓ | ✓ | ✓ | ✓ | ✓ |

*Notes*: This table employes the same controls and specifications from Table 2 in Sebahaty et al. (2020). All specifications include Driscoll-Kraay (DK) standard errors (in parenthesis). DK non-parametric standard errors are heteroskedasticity robust to cross-country and day dependences and autocorrelated consistent (up to three-day lags). $+p < .1, *p < .05, **p < .01, ***p < .001$.

Table A.8: Two-Stage Least Squares regressions — Stringency index

| ***Dependent variable: Mobility (7-day moving averages)*** | | | | | |
|---|---|---|---|---|---|
| *Panel A: First-Stage* | (1) Residential | (2) Residential | (3) Residential | (4) Residential | (5) Residential |
| Rainfall (7-day moving averages) | 1.875*** | 1.762*** | 1.563*** | 1.689*** | 1.603*** |
| | (0.358) | (0.348) | (0.333) | (0.325) | (0.304) |
| Log (Real GDP per capita) | 4.182*** | 3.934*** | 3.084*** | 3.231*** | 3.205*** |
| | (0.271) | (0.299) | (0.239) | (0.242) | (0.238) |
| Tax revenue (% of GDP) | 0.383*** | 0.383*** | 0.353*** | 0.349*** | 0.361*** |
| | (0.026) | (0.026) | (0.025) | (0.025) | (0.024) |
| GINI index (World Bank estimate) | 0.082*** | 0.064*** | 0.071*** | 0.097*** | 0.076*** |
| | (0.009) | (0.008) | (0.009) | (0.009) | (0.012) |
| Hospital beds (per 1,000 people) | 0.035 | 0.035 | 0.389*** | 0.437*** | 0.361*** |
| | (0.070) | (0.068) | (0.073) | (0.070) | (0.073) |
| Population ages 65 and above (% of total population) | -0.698*** | -0.670*** | -0.837*** | -0.898*** | -0.909*** |
| | (0.052) | (0.058) | (0.063) | (0.057) | (0.055) |
| Urban population (% of total population) | -0.050*** | -0.047*** | -0.034*** | -0.038*** | -0.026*** |
| | (0.005) | (0.005) | (0.005) | (0.006) | (0.005) |
| Log (Population density) | 0.748*** | 0.685*** | 0.625*** | 0.646*** | 0.576*** |
| | (0.098) | (0.106) | (0.105) | (0.099) | (0.102) |
| Death rate (per 100,000) | -0.002* | | | -0.004*** | -0.003*** |
| | (0.001) | | | (0.001) | (0.001) |
| Adoption density | | 0.116*** | | 0.135*** | 0.097*** |
| | | (0.016) | | (0.012) | (0.013) |
| Electoral democracy index | | | 7.104*** | 7.901*** | -0.217 |
| | | | (0.617) | (0.591) | (1.776) |
| Adoption density × Electoral democracy index | | | | | 0.080*** |
| | | | | | (0.016) |
| ***Dependent variable: Stringency index*** | | | | | |
| *Panel B: Second-Stage* | (1) | (2) | (3) | (4) | (5) |
| Residential (7-day moving averages) | 2.170*** | 2.203*** | 2.240*** | 2.230*** | 2.279*** |
| | (0.423) | (0.457) | (0.492) | (0.486) | (0.508) |
| Log (Real GDP per capita) | -2.919+ | -2.905 | -2.738+ | -2.571+ | -2.705+ |
| | (1.765) | (1.813) | (1.520) | (1.523) | (1.580) |
| Tax revenue (% of GDP) | -0.769*** | -0.798*** | -0.801*** | -0.771*** | -0.800*** |
| | (0.166) | (0.176) | (0.176) | (0.170) | (0.180) |
| GINI index (World Bank estimate) | 0.047 | 0.104* | 0.099* | 0.032 | 0.047 |
| | (0.046) | (0.046) | (0.048) | (0.053) | (0.056) |
| Hospital beds (per 1,000 people) | -2.053*** | -2.046*** | -2.180*** | -2.309*** | -2.257*** |
| | (0.214) | (0.209) | (0.245) | (0.256) | (0.259) |
| Population ages 65 and above (% of total population) | -0.370 | -0.354 | -0.269 | -0.201 | -0.146 |
| | (0.246) | (0.256) | (0.348) | (0.372) | (0.394) |
| Urban population (% of total population) | -0.040 | -0.031 | -0.034 | -0.045+ | -0.055* |
| | (0.030) | (0.031) | (0.027) | (0.027) | (0.024) |
| Log (Population density) | -1.264** | -1.357** | -1.365** | -1.244** | -1.207** |
| | (0.425) | (0.419) | (0.417) | (0.420) | (0.402) |
| Death rate (per 100,000) | 0.014*** | | | 0.015*** | 0.015*** |
| | (0.002) | | | (0.002) | (0.002) |
| Adoption density | | -0.080 | | -0.094 | -0.063 |
| | | (0.060) | | (0.070) | (0.056) |
| Electoral democracy index | | | -2.630 | -4.983 | 2.540 |
| | | | (4.137) | (4.571) | (5.658) |
| Adoption density × Electoral democracy index | | | | | -0.078 |
| | | | | | (0.061) |
| First-stage C-D F-stat | 50.387 | 47.480 | 36.639 | 44.411 | 40.326 |
| First-stage K-P F-stat | 27.452 | 25.594 | 22.096 | 26.962 | 27.812 |
| Country-days | 192 | 192 | 192 | 192 | 192 |
| N | 7771 | 8011 | 8011 | 7771 | 7771 |
| Region fixed effects | ✓ | ✓ | ✓ | ✓ | ✓ |
| Day fixed effects | ✓ | ✓ | ✓ | ✓ | ✓ |

*Notes*: This table employs the same controls and specifications from Table 2 in Sebahaty et al. (2020). All specifications include Driscoll-Kraay (DK) standard errors (in parenthesis). DK non-parametric standard errors are heteroskedasticity robust to cross-country and day dependences and autocorrelated consistent (up to three-day lags). $+p < .1, *p < .05, **p < .01, ***p < .001$.

Table A.9: Two-Stage Least Squares regressions — Stringency index — alternative moving averages

| | (1) | (2) | (3) |
|---|---|---|---|
| ***Dependent variable: Stringency index; Instrument: Rainfall (7-day moving averages)*** | | | |
| Residential (5-day moving averages) | 1.875** | | |
| | (0.608) | | |
| Residential (10-day moving averages) | | 1.888*** | |
| | | (0.556) | |
| Residential (14-day moving averages) | | | 1.693** |
| | | | (0.606) |
| Level of Democracy (Freedom House/Imputed Polity) | 1.353** | 1.370** | 1.540** |
| | (0.509) | (0.446) | (0.483) |
| ICRG Indicator of Quality of Government | -45.463*** | -45.822*** | -44.422*** |
| | (4.269) | (4.058) | (4.436) |
| Log (Confirmed cases) | 0.169 | 0.077 | 0.216 |
| | (0.618) | (0.595) | (0.652) |
| Log (Real GDP per capita) | 1.692 | 1.608 | 1.984+ |
| | (1.142) | (1.055) | (1.132) |
| Population density (people per sq. km of land area) | 0.006*** | 0.005*** | 0.005*** |
| | (0.001) | (0.001) | (0.001) |
| Population ages 65 and above (% of total population) | -1.085** | -1.131*** | -1.293*** |
| | (0.389) | (0.341) | (0.374) |
| Trade (% of GDP) | -0.072*** | -0.070*** | -0.066*** |
| | (0.014) | (0.013) | (0.014) |
| Hospital beds (per 1,000 people) | -1.408*** | -1.386*** | -1.305*** |
| | (0.221) | (0.179) | (0.172) |
| SARS | 0.593*** | 0.580*** | 0.560*** |
| | (0.034) | (0.032) | (0.034) |
| Log (Airports) | 1.433** | 1.500*** | 1.424** |
| | (0.458) | (0.438) | (0.486) |
| Adoption density | -0.012 | -0.003 | 0.021 |
| | (0.057) | (0.058) | (0.066) |
| Historical rainfall | 1.802+ | 1.584 | 1.809+ |
| | (0.988) | (0.998) | (0.988) |
| First-stage C-D F-stat | 17.861 | 17.636 | 16.610 |
| First-stage K-P F-stat | 9.840 | 10.587 | 11.175 |
| Country-days | 193 | 188 | 184 |
| N | 10994 | 10835 | 10707 |
| Region fixed effects | ✓ | ✓ | ✓ |
| Day fixed effects | ✓ | ✓ | ✓ |

*Notes*: All specifications include Driscoll-Kraay (DK) standard errors (in parenthesis). DK non-parametric standard errors are heteroskedasticity robust to cross-country and day dependences and autocorrelated consistent (up to three-day lags). $+p < .1, *p < .05, **p < .01, ***p < .001$.

Table A.10: Two-Stage Least Squares regressions — Stringency index — dropping extreme cases of rainfalls

| | (1) | (2) | (3) |
|---|---|---|---|
| | > 1% | > 5% | > 10% |
| ***Dependent variable: Stringency index; Instrument: Rainfall (7-day moving averages)*** | | | |
| Residential (7-day moving averages) | 1.873** | 2.105*** | 2.969*** |
| | (0.571) | (0.504) | (0.534) |
| Level of Democracy (Freedom House/Imputed Polity) | 1.359** | 1.437*** | 1.031* |
| | (0.473) | (0.401) | (0.428) |
| ICRG Indicator of Quality of Government | -45.551*** | -45.236*** | -50.160*** |
| | (4.069) | (3.885) | (3.557) |
| Log (Confirmed cases) | 0.135 | 0.119 | -0.620 |
| | (0.598) | (0.573) | (0.543) |
| Log (Real GDP per capita) | 1.644 | 0.902 | -1.111 |
| | (1.081) | (0.973) | (1.118) |
| Population density (people per sq. km of land area) | 0.005*** | 0.007*** | 0.008*** |
| | (0.001) | (0.001) | (0.001) |
| Population ages 65 and above (% of total population) | -1.113** | -1.149*** | -0.753* |
| | (0.362) | (0.318) | (0.318) |
| Trade (% of GDP) | -0.070*** | -0.079*** | -0.103*** |
| | (0.013) | (0.014) | (0.016) |
| Hospital beds (per 1,000 people) | -1.402*** | -1.297*** | -1.320*** |
| | (0.203) | (0.209) | (0.218) |
| SARS | 0.585*** | 0.617*** | 0.678*** |
| | (0.033) | (0.040) | (0.046) |
| Log (Airports) | 1.455** | 1.684*** | 2.172*** |
| | (0.446) | (0.369) | (0.404) |
| Adoption density | -0.007 | -0.023 | -0.099 |
| | (0.057) | (0.058) | (0.069) |
| Historical rainfall | 1.681+ | 0.674 | 0.206 |
| | (1.002) | (0.983) | (1.480) |
| First-stage C-D F-stat | 18.306 | 31.274 | 40.116 |
| First-stage K-P F-stat | 10.230 | 17.885 | 25.682 |
| Country-days | 191 | 191 | 191 |
| N | 10920 | 10314 | 9470 |
| Region fixed effects | ✓ | ✓ | ✓ |
| Day fixed effects | ✓ | ✓ | ✓ |

*Notes*: All specifications include Driscoll-Kraay (DK) standard errors (in parenthesis). DK non-parametric standard errors are heteroskedasticity robust to cross-country and day dependences and autocorrelated consistent (up to three-day lags). $+p < .1, *p < .05, **p < .01, ***p < .001$.

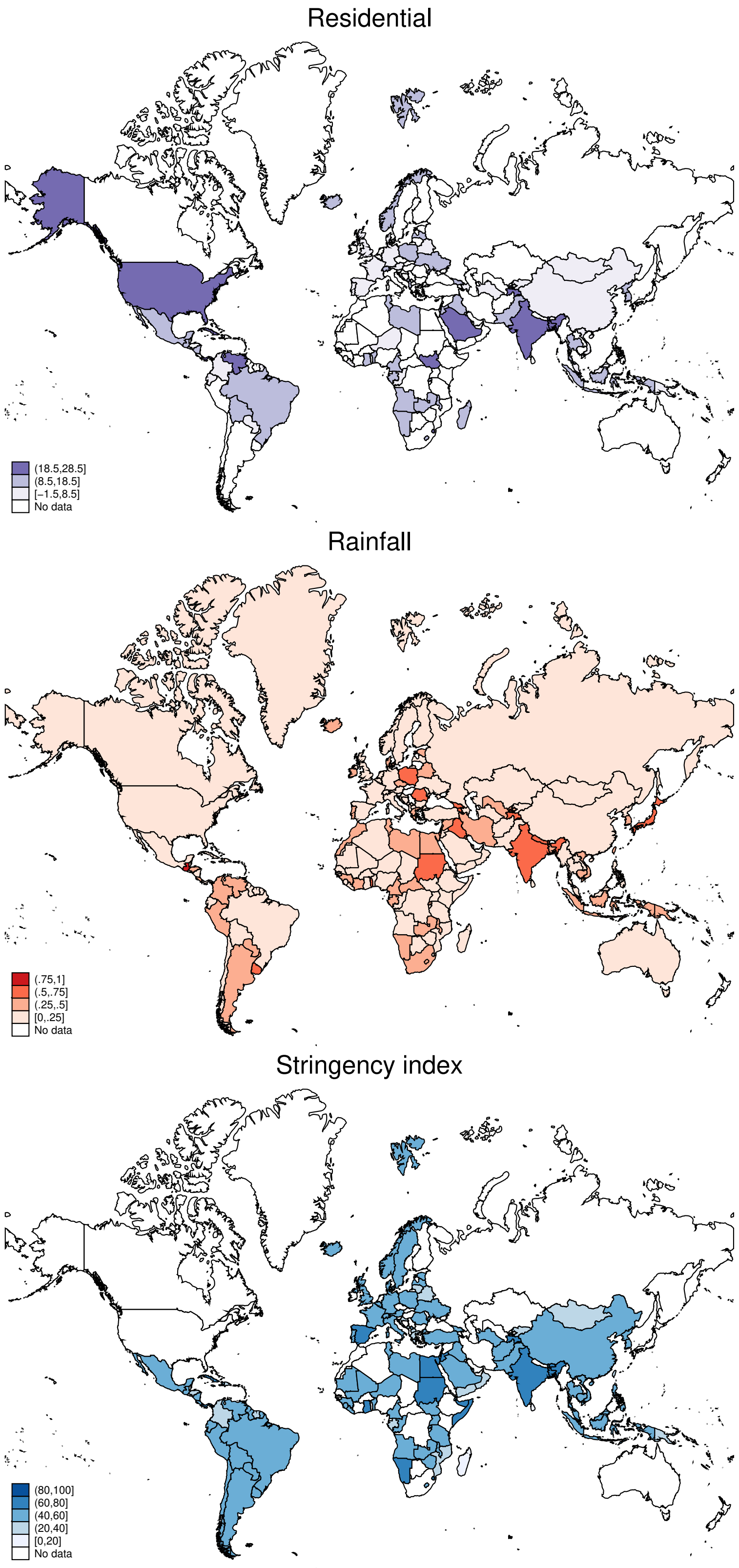


Figure A.1: Geographic Variation in Mobility, Rainfall and Stringency index (means). The maps show territories and their respective sovereign states, even if the last ones are not in our sample.

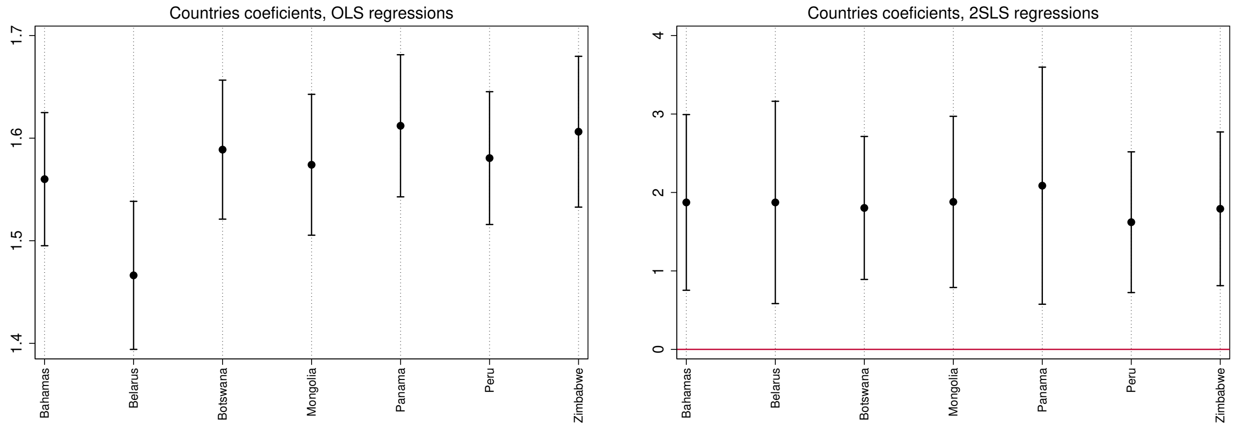


Figure A.2: Leave-One-Out Checks for the OLS and 2SLS models. Each estimate is based on a sample that omits countries with extreme low and high values on Walking on the x-axis. Dots are coefficients; bars are 95% CIs. The dependent variable is the Stringency index. All models include the same controls as column 3 from Table 1 and column 2 from Table 2. All specifications include Driscoll-Kraay (DK) standard errors (in parenthesis). DK non-parametric standard errors are heteroskedasticity robust to cross-country and day dependences and autocorrelated consistent (up to three-day lags).

Table A.11: Two-Stage Least Squares regressions — Stringency index — dropping extreme cases of population

| | (1) | (2) | (3) |
|---|---|---|---|
| | > 1% | > 5% | > 10% |
| ***Dependent variable: Stringency index; Instrument: Rainfall (7-day moving averages)*** | | | |
| Residential (7-day moving averages) | 1.983*** | 2.005** | 2.542** |
| | (0.581) | (0.621) | (0.794) |
| Level of Democracy (Freedom House/Imputed Polity) | 1.260* | 1.439* | 1.429* |
| | (0.492) | (0.606) | (0.602) |
| ICRG Indicator of Quality of Government | -46.565*** | -46.774*** | -43.840*** |
| | (4.345) | (3.812) | (1.170) |
| Log (Confirmed cases) | 0.024 | -0.194 | -0.749 |
| | (0.614) | (0.880) | (1.318) |
| Log (Real GDP per capita) | 1.463 | 1.577 | -0.686 |
| | (1.077) | (1.106) | (1.766) |
| Population density (people per sq. km of land area) | 0.006*** | 0.004*** | 0.007*** |
| | (0.001) | (0.001) | (0.001) |
| Population ages 65 and above (% of total population) | -1.031** | -1.065* | -1.099** |
| | (0.380) | (0.489) | (0.386) |
| Trade (% of GDP) | -0.073*** | -0.077*** | -0.123*** |
| | (0.014) | (0.013) | (0.028) |
| Hospital beds (per 1,000 people) | -1.430*** | -1.133*** | -0.828** |
| | (0.211) | (0.164) | (0.305) |
| SARS | 0.590*** | 0.562*** | 1.462* |
| | (0.033) | (0.029) | (0.592) |
| Log (Airports) | 1.497*** | 1.419*** | 1.870*** |
| | (0.424) | (0.142) | (0.256) |
| Adoption density | -0.013 | -0.071 | -0.129 |
| | (0.058) | (0.079) | (0.103) |
| Historical rainfall | 1.441 | 0.217 | 0.469 |
| | (1.034) | (1.170) | (1.216) |
| First-stage C-D F-stat | 18.373 | 22.156 | 14.818 |
| First-stage K-P F-stat | 10.570 | 13.140 | 8.453 |
| Country-days | 191 | 191 | 191 |
| N | 10736 | 9874 | 8990 |
| Region fixed effects | ✓ | ✓ | ✓ |
| Day fixed effects | ✓ | ✓ | ✓ |

*Notes*: All specifications include Driscoll-Kraay (DK) standard errors (in parenthesis). DK non-parametric standard errors are heteroskedasticity robust to cross-country and day dependences and autocorrelated consistent (up to three-day lags). $+p < .1, *p < .05, **p < .01, ***p < .001$.

Table A.12: Two-Stage Least Squares regressions — Stringency index — dropping large countries

| | (1) | (2) | (3) |
|---|---|---|---|
| ***Dependent variable: Stringency index; Instrument: Rainfall (7-day moving averages)*** | | | |
| Residential (7-day moving averages) | 1.861*** | 2.923+ | 3.751* |
| | (0.564) | (1.559) | (1.848) |
| Level of Democracy (Freedom House/Imputed Polity) | 1.402** | 0.257 | -0.300 |
| | (0.477) | (1.420) | (1.724) |
| ICRG Indicator of Quality of Government | -44.694*** | -54.813*** | -61.090*** |
| | (4.122) | (10.021) | (11.767) |
| Log (Confirmed cases) | 0.073 | -1.157 | -1.981 |
| | (0.620) | (1.830) | (2.193) |
| Log (Real GDP per capita) | 1.602 | 0.293 | -1.621 |
| | (1.044) | (2.786) | (3.190) |
| Population density (people per sq. km of land area) | 0.005*** | 0.006*** | 0.007** |
| | (0.001) | (0.002) | (0.002) |
| Population ages 65 and above (% of total population) | -1.138** | -0.334 | 0.450 |
| | (0.358) | (1.056) | (1.208) |
| Trade (% of GDP) | -0.069*** | -0.077* | -0.076+ |
| | (0.014) | (0.030) | (0.039) |
| Hospital beds (per 1,000 people) | -1.369*** | -3.045*** | -3.368*** |
| | (0.209) | (0.781) | (0.975) |
| SARS | 0.579*** | 0.605*** | 0.609*** |
| | (0.034) | (0.046) | (0.061) |
| Log (Airports) | 1.402** | 1.627** | 1.913* |
| | (0.464) | (0.631) | (0.759) |
| Adoption density | -0.003 | -0.084 | -0.139 |
| | (0.056) | (0.134) | (0.155) |
| Historical rainfall | 1.629 | 0.751 | 0.255 |
| | (0.998) | (1.915) | (2.394) |
| First-stage C-D F-stat | 19.473 | 3.305 | 3.745 |
| First-stage K-P F-stat | 10.838 | 1.958 | 2.265 |
| Country-days | 191 | 191 | 191 |
| N | 10740 | 10200 | 10034 |
| Region fixed effects | ✓ | ✓ | ✓ |
| Day fixed effects | ✓ | ✓ | ✓ |

*Notes*: All specifications include Driscoll-Kraay (DK) standard errors (in parenthesis). DK non-parametric standard errors are heteroskedasticity robust to cross-country and day dependences and autocorrelated consistent (up to three-day lags). The regressions progressively exclude countries with large areas based on the ranking of the first ten countries of *The World Factbook*. The first column drops Russia, and China. The third drops Russia, China, Brazil, India, and Argentina. The fourth drops. Finally, the fifth column drops Russia, Antarctica, China, Brazil, India, Argentina, and Kazakhstan. $+p < .1, *p < .05, **p < .01, ***p < .001$.

Table A.13: Two-Stage Least Squares regressions — Stringency index — COVID-19 "unrelated" conflicts

| | (1) | (2) | (3) | (4) | (5) |
|---|---|---|---|---|---|
| ***Dependent variable: Stringency index; Instrument: Rainfall (7-day moving averages)*** | | | | | |
| Fatalities (7-day mov. avgs) - not just C-19 | -246.637 | | | | |
| | (11911.911) | | | | |
| Protests (7-day mov. avgs) - not just C-19 | | -2.382 | | | |
| | | (1.644) | | | |
| Riots (7-day mov. avgs) - not just C-19 | | | -5.160 | | |
| | | | (3.159) | | |
| Viol. civilians (7-day mov. avgs) - not just C-19 | | | | -19.698 | |
| | | | | (40.081) | |
| Battles (7-day mov. avgs) - not just C-19 | | | | | 3.362+ |
| | | | | | (1.960) |
| Level of Democracy (Freedom House/Imputed Polity) | 108.140 | 3.223*** | 3.524*** | 2.238* | 1.704*** |
| | (5094.478) | (0.498) | (0.671) | (1.059) | (0.484) |
| ICRG Indicator of Quality of Government | -3310.933 | -22.013* | -12.495 | -52.247 | -33.338*** |
| | (158281.245) | (9.711) | (12.569) | (39.181) | (5.501) |
| Log (Confirmed cases) | 108.273 | 2.617*** | 2.070*** | 4.124 | 1.507* |
| | (5107.500) | (0.298) | (0.411) | (3.254) | (0.683) |
| Log (Real GDP per capita) | -364.170 | 5.813** | 7.625** | -4.159 | 11.165* |
| | (17780.390) | (2.034) | (2.662) | (16.961) | (4.501) |
| Population density (people per sq. km of land area) | 1.404 | -0.014 | 0.007 | 0.123 | -0.002 |
| | (67.852) | (0.009) | (0.007) | (0.254) | (0.003) |
| Population ages 65 and above (% of total population) | -109.347 | -4.525*** | -4.384*** | -7.964 | -1.873 |
| | (5094.234) | (0.532) | (0.378) | (8.441) | (1.188) |
| Trade (% of GDP) | 28.349 | 0.131 | 0.014 | 1.487 | -0.150** |
| | (1372.511) | (0.137) | (0.051) | (3.157) | (0.054) |
| Hospital beds (per 1,000 people) | -173.381 | 2.955*** | 1.201 | -5.138 | 1.181 |
| | (8480.943) | (0.835) | (1.111) | (14.973) | (0.892) |
| SARS | -48.547 | -0.044 | 0.286 | -4.649 | 0.250 |
| | (2373.096) | (0.479) | (0.284) | (10.616) | (0.263) |
| Log (Airports) | 660.815 | 1.035 | -0.913 | 37.929 | -5.839*** |
| | (32090.180) | (3.452) | (1.846) | (84.744) | (1.589) |
| Adoption density | -2.775 | -0.010 | 0.321*** | 0.302*** | 0.273*** |
| | (147.618) | (0.196) | (0.037) | (0.075) | (0.029) |
| Historical rainfall | 127.286 | 5.350 | -0.449 | 9.842 | -2.524 |
| | (6177.602) | (3.773) | (1.415) | (21.915) | (2.251) |
| First-stage C-D F-stat | 0.001 | 6.235 | 31.098 | 0.355 | 8.907 |
| First-stage K-P F-stat | 0.000 | 2.704 | 10.049 | 0.193 | 4.016 |
| Country-days | 236 | 236 | 236 | 236 | 236 |
| N | 3702 | 3702 | 3702 | 3702 | 3702 |
| Region fixed effects | ✓ | ✓ | ✓ | ✓ | ✓ |
| Day fixed effects | ✓ | ✓ | ✓ | ✓ | ✓ |

*Notes*: All specifications include Driscoll-Kraay (DK) standard errors (in parenthesis). DK non-parametric standard errors are heteroskedasticity robust to cross-country and day dependences and autocorrelated consistent (up to three-day lags). $+p < .1, *p < .05, **p < .01, ***p < .001$.

Table A.14: Two-Stage Least Squares regressions — Stringency index — Squared IVs

| ***Instruments: Rainfall measures (7-day moving averages)*** | | | |
|---|---|---|---|
| | (1) Rainfall (> 0.1 inch) | (2) PRCP (hundreth of inches) | (3) PRCP (hundreth of inches, IHS) |
| *Panel A: First-Stage* | Residential | Residential | Residential |
| Rainfall (7-day moving averages) | 3.418*** | | |
| | (0.934) | | |
| Rainfall (7-day moving averages) squared | -3.056* | | |
| | (1.246) | | |
| Precipitation (hundreth of inches) (7-day moving averages) | | 12.687*** | |
| | | (2.646) | |
| PRCP (hundreth of inches) squared | | -11.234** | |
| | | (3.561) | |
| Precipitation (hundreth of inches, IHS) (7-day moving averages) | | | 22.145*** |
| | | | (4.538) |
| PRCP (hundreth of inches, IHS) squared | | | -21.773*** |
| | | | (5.582) |
| Level of Democracy (Freedom House/Imputed Polity) | 0.807*** | 0.821*** | 0.824*** |
| | (0.040) | (0.040) | (0.040) |
| ICRG Indicator of Quality of Government | 6.329*** | 6.532*** | 6.499*** |
| | (0.493) | (0.511) | (0.512) |
| Log (Confirmed cases) | 0.926*** | 0.948*** | 0.950*** |
| | (0.057) | (0.056) | (0.056) |
| Log (Real GDP per capita) | 1.988*** | 1.936*** | 1.938*** |
| | (0.123) | (0.121) | (0.122) |
| Population density (people per sq. km of land area) | -0.001*** | -0.002*** | -0.002*** |
| | (0.000) | (0.000) | (0.000) |
| Population ages 65 and above (% of total population) | -0.701*** | -0.686*** | -0.684*** |
| | (0.032) | (0.032) | (0.032) |
| Trade (% of GDP) | 0.021*** | 0.022*** | 0.022*** |
| | (0.002) | (0.002) | (0.002) |
| Hospital beds (per 1,000 people) | 0.319*** | 0.340*** | 0.344*** |
| | (0.048) | (0.051) | (0.051) |
| SARS | -0.058*** | -0.054*** | -0.054*** |
| | (0.013) | (0.012) | (0.012) |
| Log (Airports) | -0.634*** | -0.656*** | -0.662*** |
| | (0.060) | (0.063) | (0.062) |
| Adoption density | 0.083*** | 0.086*** | 0.087*** |
| | (0.009) | (0.009) | (0.009) |
| Historical rainfall | 0.762 | 0.330 | 0.273 |
| | (0.464) | (0.441) | (0.443) |
| ***Dependent variable: Stringency index*** | | | |
| *Panel B: Second-Stage* | (1) | (2) | (3) |
| Residential (7-day moving averages) | 3.141*** | 2.277*** | 2.352*** |
| | (0.580) | (0.257) | (0.308) |
| Level of Democracy (Freedom House/Imputed Polity) | 0.328 | 1.030*** | 0.969*** |
| | (0.468) | (0.230) | (0.266) |
| ICRG Indicator of Quality of Government | -53.959*** | -48.231*** | -48.727*** |
| | (3.968) | (2.307) | (2.593) |
| Log (Confirmed cases) | -1.072+ | -0.250 | -0.321 |
| | (0.611) | (0.237) | (0.273) |
| Log (Real GDP per capita) | -0.784 | 0.870+ | 0.727 |
| | (1.162) | (0.464) | (0.533) |
| Population density (people per sq. km of land area) | 0.007*** | 0.006*** | 0.006*** |
| | (0.001) | (0.001) | (0.001) |
| Population ages 65 and above (% of total population) | -0.255 | -0.839*** | -0.789*** |
| | (0.367) | (0.186) | (0.209) |
| Trade (% of GDP) | -0.097*** | -0.079*** | -0.080*** |
| | (0.013) | (0.009) | (0.009) |
| Hospital beds (per 1,000 people) | -1.803*** | -1.530*** | -1.554*** |
| | (0.240) | (0.206) | (0.209) |
| SARS | 0.657*** | 0.608*** | 0.612*** |
| | (0.035) | (0.030) | (0.031) |
| Log (Airports) | 2.315*** | 1.729*** | 1.780*** |
| | (0.469) | (0.224) | (0.250) |
| Adoption density | -0.114+ | -0.041 | -0.047 |
| | (0.065) | (0.030) | (0.033) |
| Historical rainfall | 0.283 | 1.236 | 1.153 |
| | (1.147) | (0.830) | (0.881) |
| First-stage C-D F-stat | 20.108 | 38.831 | 35.433 |
| First-stage K-P F-stat | 12.799 | 30.537 | 26.677 |
| Country-days | 191 | 191 | 191 |
| N | 10920 | 10920 | 10920 |
| Region fixed effects | ✓ | ✓ | ✓ |
| Day fixed effects | ✓ | ✓ | ✓ |

*Notes*: All specifications include Driscoll-Kraay (DK) standard errors (in parenthesis). DK non-parametric standard errors are heteroskedasticity robust to cross-country and day dependences and autocorrelated consistent (up to three-day lags). $+p < .1, *p < .05, **p < .01, ***p < .001$.

Table A.15: Two-Stage Least Squares regressions — Replication with strongest IVs

| ***Dependent variable: Stringency index*** | | | | |
|---|---|---|---|---|
| ***Instrumental variables:*** | PRCP (hundreth of inches) | | PRCP (hundreth of inches, IHS) | |
| | (1) | (2) | (3) | (4) |
| Residential (7-day moving averages) | 2.591*** | 2.359*** | 2.696*** | 2.430*** |
| | (0.271) | (0.276) | (0.290) | (0.311) |
| Level of Democracy (Freedom House/Imputed Polity) | 0.717** | 0.964*** | 0.630* | 0.906*** |
| | (0.245) | (0.242) | (0.258) | (0.266) |
| ICRG Indicator of Quality of Government | -49.975*** | -48.771*** | -50.669*** | -49.244*** |
| | (2.506) | (2.492) | (2.684) | (2.728) |
| Log (Confirmed cases) | -0.554* | -0.328 | -0.655* | -0.395 |
| | (0.269) | (0.281) | (0.296) | (0.320) |
| Log (Real GDP per capita) | 0.205 | 0.714 | 0.007 | 0.578 |
| | (0.534) | (0.504) | (0.554) | (0.551) |
| Population density (people per sq. km of land area) | 0.006*** | 0.006*** | 0.006*** | 0.006*** |
| | (0.001) | (0.001) | (0.001) | (0.001) |
| Population ages 65 and above (% of total population) | -0.600** | -0.784*** | -0.529* | -0.736*** |
| | (0.204) | (0.201) | (0.212) | (0.217) |
| Trade (% of GDP) | -0.089*** | -0.081*** | -0.091*** | -0.082*** |
| | (0.009) | (0.009) | (0.010) | (0.010) |
| Hospital beds (per 1,000 people) | -1.632*** | -1.556*** | -1.667*** | -1.578*** |
| | (0.218) | (0.210) | (0.221) | (0.212) |
| SARS | 0.626*** | 0.613*** | 0.633*** | 0.617*** |
| | (0.030) | (0.030) | (0.031) | (0.031) |
| Log (Airports) | 1.907*** | 1.784*** | 1.979*** | 1.833*** |
| | (0.241) | (0.242) | (0.257) | (0.266) |
| Adoption density | | -0.048 | | -0.054 |
| | | (0.033) | | (0.036) |
| Historical rainfall | | 1.146 | | 1.067 |
| | | (0.867) | | (0.912) |
| First-stage C-D F-stat | 70.439 | 68.709 | 58.633 | 57.494 |
| First-stage K-P F-stat | 42.064 | 39.007 | 33.825 | 30.559 |
| Country-days | 192 | 191 | 192 | 191 |
| N | 11009 | 10920 | 11009 | 10920 |
| Region fixed effects | ✓ | ✓ | ✓ | ✓ |
| Day fixed effects | ✓ | ✓ | ✓ | ✓ |

*Notes*: All specifications include Driscoll-Kraay (DK) standard errors (in parenthesis). DK non-parametric standard errors are heteroskedasticity robust to cross-country and day dependences and autocorrelated consistent (up to three-day lags). The full Table with the coefficients of the First-stage regressions are reported in the Appendix. $+p < .1, *p < .05, **p < .01, ***p < .001$.

Table A.16: Two-Stage Least Squares regressions — Alternative IVs

| | Max. wind speed (7-day moving averages) | Rapid fluct. of wind speed (7-day moving averages) | Visibility (7-day moving averages) |
|---|---|---|---|
| | (1) | (2) | (3) |
| ***Dependent variable: Stringency index*** | | | |
| Residential (7-day moving averages) | 5.524*** | 2.108*** | 1.971*** |
| | (0.408) | (0.547) | (0.362) |
| Level of Democracy (Freedom House/Imputed Polity) | 0.305 | 0.982** | 1.068*** |
| | (0.799) | (0.379) | (0.244) |
| ICRG Indicator of Quality of Government | -70.033*** | -44.702*** | -43.579*** |
| | (10.178) | (5.129) | (3.576) |
| Log (Confirmed cases) | -3.820*** | -0.056 | 0.088 |
| | (0.830) | (0.573) | (0.379) |
| Log (Real GDP per capita) | 7.978*** | 1.376 | 1.659* |
| | (1.924) | (0.922) | (0.672) |
| Population density (people per sq. km of land area) | 0.104** | 0.007*** | 0.007*** |
| | (0.032) | (0.001) | (0.001) |
| Population ages 65 and above (% of total population) | 0.477 | -0.808** | -0.886*** |
| | (0.606) | (0.304) | (0.202) |
| Trade (% of GDP) | -0.146*** | -0.092*** | -0.090*** |
| | (0.025) | (0.011) | (0.007) |
| Hospital beds (per 1,000 people) | -0.158 | -1.686*** | -1.685*** |
| | (0.866) | (0.139) | (0.148) |
| SARS | 1.433** | 0.614*** | 0.607*** |
| | (0.487) | (0.030) | (0.021) |
| Log (Airports) | 7.783*** | 1.641*** | 1.527*** |
| | (1.203) | (0.442) | (0.284) |
| Adoption density | -0.394*** | -0.042 | -0.033 |
| | (0.097) | (0.050) | (0.039) |
| Historical rainfall | -7.730+ | 1.651 | 1.830* |
| | (4.362) | (1.076) | (0.811) |
| First-stage C-D F-stat | 255.004 | 33.815 | 52.229 |
| First-stage K-P F-stat | 92.526 | 19.715 | 39.933 |
| Country-days | 191 | 191 | 191 |
| N | 2918 | 11178 | 11189 |
| Region fixed effects | ✓ | ✓ | ✓ |
| Day fixed effects | ✓ | ✓ | ✓ |

*Notes*: All specifications include Driscoll-Kraay (DK) standard errors (in parenthesis). DK non-parametric standard errors are heteroskedasticity robust to cross-country and day dependences and autocorrelated consistent (up to three-day lags). $+p < .1, *p < .05, **p < .01, ***p < .001$.

## A.2 Sensitivity analysis for 2SLS

To illustrate the robustness of our IV results to limited violations of the exclusion restriction, we perform the test proposed by Conley, Hansen and Rossi [11]. In this exercise, we allow "Rainfall (7-day moving averages)" to have a direct effect on "Stringency index" and then re-estimate the IV coefficient of "Residential (7-day moving averages)." We let the direct effect take any value between zero and the coefficient of the reduced form[1]: for each of these direct effects, we calculate the union of the 95% confidence intervals of the IV coefficient. In Figure A.3 we plot these confidence intervals (y-axis) against the assumed direct effect of the instrument (x-axis). The figure employs our baseline estimate, clustering at country-days. The blue vertical line flags the value of the reduced form coefficients. The green vertical line represents the value that brings our estimates to zero. To read the results of this test, we compare the reduced form coefficients to the value of the direct effect where the union of confidence intervals crosses the zero. We find that the direct effect of "Rainfall (7-day moving averages)" on "Stringency index" would have to account for 40% of the overall reduced form effect before the estimated coefficient becomes insignificant. As "Rainfall (7-day moving averages)" is unlikely to be strong correlated with "Stringency index", we consider such large direct effect unlikely.

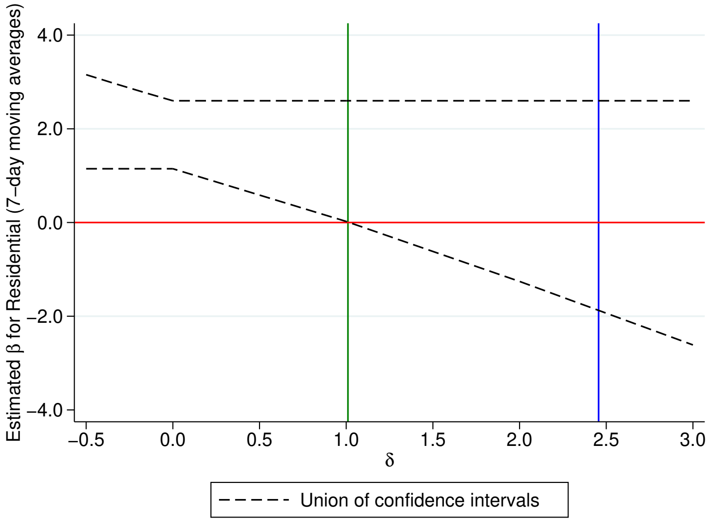


Figure A.3: Union of confidence intervals of the IV estimates (y-axis) when the exclusion restriction is violated (x-axis). Blue vertical line: point estimate of the reduced form coefficient (column 3 of Table A.5). Green vertical line: point estimate that brings our estimates to zero.

[1]The coefficient of 2SLS estimates are identical to the ratio of the reduced form point estimate to the first stage coefficient. Therefore, confounders can bring the instrumental variable point estimate to zero if they also bring the reduced-form coefficient to zero [1, 9].

Table A.17: Variables

| Variable name | Definition | Source |
|---|---|---|
| **Dependent variables** | | |
| Stringency Index | A composite index between 0 and 100, where a higher score indicates more stringent government responses to COVID-19. The index is created based on the ordinal values of OxCGRT policy variables C1–C8 and H1. The score is rescaled for each measure by their maximum value to create a score between 0 and 100, with a missing value contributing 0. The composite Stringency Index is the average of these nine re-scaled scores | Hale et al. [17] |
| **Independent variables** | | |
| Residential mobility | Visits and length of stay at residential places, compared to a baseline, calculated using aggregate data from Google Maps. Google records wait times and visit durations for users who opted in to Google Location History. The baseline is the median value, for the corresponding day of the week, during the 5-week period of 6 January – 6 February, 2020. | Google LLC [15] |
| Walking | The relative volume of direction requests in Apple Maps per country, compared to a baseline volume on 13 January 2020. Data is collected on a daily basis and records requests when 'walking' is selected as transportation type in Apple Maps. | Apple [2] |
| Retail and recreation | Visits and length of stay at retail and recreation locations, compared to a baseline, calculated using aggregate data from Google Maps. Google records wait times and visit durations for users who opted in to Google Location History. The baseline is the median value, for the corresponding day of the week, during the 5-week period of 6 January – 6 February, 2020. | Google LLC [15] |
| Grocery and pharmacy | Visits and length of stay at groceries and pharmacies, compared to a baseline, calculated using aggregate data from Google Maps. Google records wait times and visit durations for users who opted in to Google Location History. The baseline is the median value, for the corresponding day of the week, during the 5-week period of 6 January – 6 February, 2020. | Google LLC [15] |
| Protests | Non-violent demonstrations as recorded by the Armed Conflict Location & Event Data Project (ACLED) at the country-level, updated daily, which might involve unorganized action by members of society, and might have been met by (excessive) force from governments' side. Besides all protests, we also use a curated subset of the data, containing protests directly related to the coronavirus pandemic, obtained from ACLED's COVID-19 Disorder Tracker. | Raleigh et al. [24] |

Table A.18: Variables

| Variable name | Definition | Source |
|---|---|---|
| **Independent variables** | | |
| Riots | Violent demonstrations recorded by the Armed Conflict Location & Event Data Project (ACLED) at the country-level, updated daily. The variable might capture spontaneous action by unorganized, unaffiliated members of society; clashes between protesters and police, as well as mob violence. Besides all riots, we also use a curated subset of the data, containing riots directly related to the coronavirus pandemic, obtained from ACLED's COVID-19 Disorder Tracker. | Raleigh et al. [24] |
| **Instrumental variables** | | |
| Rainfall | Precipitation amount (in .01 inches), measured daily, as recorded in the Global Surface Summary of the Day (GSOD) database. We add observations from all weather stations within a country, take the mean value, and compute their 7-day-moving averages for each country. Alternative operationalizations (presented in the Appendix), such as | National Oceanic and Atmospheric Administration [21] |
| **Control variables** | | |
| Level of Democracy | Level of democracy on a scale from 0-10, where 0 is least democratic and 10 most democratic. Average of Freedom House (fh_pr and fh_cl) is transformed to a scale 0-10 and Polity (p_polity2) is transformed to a scale 0-10. These variables are averaged into fh_polity2. The are imputed values for countries where data on Polity is missing by regressing Polity on the average Freedom House measure [28]. | From Teorell et al. [28], based on Freedom House [13] and Marshall, Gurr and Jaggers [20] |
| Quality of Government | The ICRG Indicator of Quality of Government, scaled 0–1. Computed as the mean value of International Country Risk Guide (ICRG) subcategories *Corruption*, *Law and Order* and *Bureaucracy Quality*; originally composed of 22 variables. | From PRS [22], retrieved from Teorell et al. [28] |
| Confirmed cases | Total number of confirmed COVID-19 cases, updated daily between 22 January 2020 – 31 August 2020 | From Hale et al. [17] |
| Real GDP per capita | Real GDP per capita in 2011 US dollars, multiple benchmarks. | From Bolt et al. [5] retrieved from Teorell et al. [28] |
| Population density | People per sq. km of land area. Population counts all residents regardless of legal status or citizenship. (Year 2016) | From World Bank [34], retrieved from Teorell et al. [28] |

Table A.19: Variables

| Variable name | Definition | Source |
|---|---|---|
| **Control variables** | | |
| Population ages 65 and above | Population ages 65 and above as a percentage of the total population. Population counts all residents regardless of legal status or citizenship. (Year 2016) | From World Bank [34], retrieved from Teorell et al. [28] |
| Trade (% of GDP) | The sum of exports and imports of goods and services measured as a share of gross domestic product. | From World Bank [34], retrieved from Teorell et al. [28] |
| Hospital beds (per 1,000) | Number of hospital beds per 1,000 people, from most recent year available since 2010; data collected by Roser et al. [25] from OECD, Eurostat, World Bank, national government records and other sources. | From Roser et al. [25] |
| SARS | The number of confirmed SARS cases in the country in 2002-2003 | Based on WHO [33] |
| Number of airports | The total number of airports or airfields recognizable from the air. The runway(s) may be paved or unpaved and may include closed or abandoned installations. | From The World Factbook [29] |
| Adoption density | A measure capturing prior adoption of policies (as recorded by OxCGRT) among spatially proximate countries. We calculate a neighborhood average of the Stringency Index, borrowing the approach of Sebhatu et al. [26]. | Hale et al. [17], based on Sebhatu et al. [26] |
| Electoral democracy index | The Electoral Democracy Index (EDI) is derived from expert surveys, where the Varieties of Democracy (V-Dem) project asked scholars to rate countries along each of the 43 indicators measuring institutions of democracy. It ranges from 0 to 100, where 100 indicates that democracy is "achieved in its fullest sense", whereas 0 stands for pure dictatorship. | Coppedge et al. [12] |
| Tax revenue (% of GDP) | The share of compulsory transfers to the central government, according to IMF, World Bank and Government Finance Statistics Yearbook; OECD estimates for GDP. | From World Bank [34], retrieved from Teorell et al. [28] |
| Gini index | Gini index measures the extent to which the distribution of income among individuals or households within an economy deviates from a perfectly equal distribution on a scale from 0 to 100, where 0 represents perfect equality and 100 implies perfect inequality. | From World Bank [34], retrieved from Teorell et al. [28] |
| Urban population | Share of the total population (in %) living in urban areas, as recorded by United Nations Population Division. | From World Bank [34], retrieved from Teorell et al. [28] |
| Death rate per 100,000 | Computed from OxCGRT dataset's *confirmed deaths* variable, divided by 100,000. | From Hale et al. [17] |
| Federalism | Dichotomous variable, where 1 stands for the presence of independent sub-federal units constraining national fiscal policy. | From Teorell et al. [28] |
| Latitude / Longitude | Average longitude and latitude coordinates for all countries. | From Wang [30] |
| Subnational policies | Dichotomous variable in the OxCGRT dataset. It takes the value 0 if coronavirus policies are targeted to a specific geographical region and 1 if they apply to the whole country. | From Hale et al. [17] |

Table A.20: Two-Stage Least Squares regressions — Stringency index — federalism, latitude, and longitude controls

| | (1) | (2) | (3) |
|---|---|---|---|
| ***Dependent variable: Stringency index; Instrument: Rainfall (7-day moving averages)*** | | | |
| Residential (7-day moving averages) | 1.828** | 2.106*** | 2.056*** |
| | (0.667) | (0.473) | (0.590) |
| Level of Democracy (Freedom House/Imputed Polity) | 1.496* | 1.143*** | 1.276** |
| | (0.607) | (0.336) | (0.476) |
| ICRG Indicator of Quality of Government | -45.275*** | -48.427*** | -47.160*** |
| | (3.766) | (2.021) | (2.117) |
| Log (Confirmed cases) | 0.394 | 0.004 | 0.190 |
| | (0.838) | (0.524) | (0.744) |
| Log (Real GDP per capita) | 1.794 | 0.933 | 1.009 |
| | (1.465) | (1.048) | (1.497) |
| Population density (people per sq. km of land area) | 0.005*** | 0.005*** | 0.005*** |
| | (0.001) | (0.001) | (0.001) |
| Population ages 65 and above (% of total population) | -1.258* | -0.919** | -1.033* |
| | (0.502) | (0.346) | (0.493) |
| Trade (% of GDP) | -0.042 | -0.072*** | -0.052+ |
| | (0.038) | (0.009) | (0.030) |
| Hospital beds (per 1,000 people) | -1.352*** | -1.604*** | -1.496*** |
| | (0.202) | (0.153) | (0.168) |
| SARS | 0.533*** | 0.595*** | 0.558*** |
| | (0.074) | (0.026) | (0.055) |
| Log (Airports) | 1.782*** | 1.379*** | 1.602*** |
| | (0.215) | (0.392) | (0.206) |
| Adoption density | -0.003 | -0.027 | -0.023 |
| | (0.066) | (0.050) | (0.059) |
| Historical rainfall | 1.765+ | 1.602 | 1.681 |
| | (1.020) | (1.058) | (1.076) |
| Federalism | -6.319 | | -4.161 |
| | (5.381) | | (4.561) |
| Latitude | | -0.038 | -0.026 |
| | | (0.044) | (0.039) |
| Longitude | | 0.021 | 0.022 |
| | | (0.030) | (0.033) |
| First-stage C-D F-stat | 14.240 | 31.526 | 21.145 |
| First-stage K-P F-stat | 7.767 | 16.188 | 11.320 |
| Country-days | 191 | 191 | 191 |
| N | 10768 | 10920 | 10768 |
| Region fixed effects | ✓ | ✓ | ✓ |
| Day fixed effects | ✓ | ✓ | ✓ |

*Notes*: All specifications include Driscoll-Kraay (DK) standard errors (in parenthesis). DK non-parametric standard errors are heteroskedasticity robust to cross-country and day dependences and autocorrelated consistent (up to three-day lags). $+p < .1, *p < .05, **p < .01, ***p < .001$.

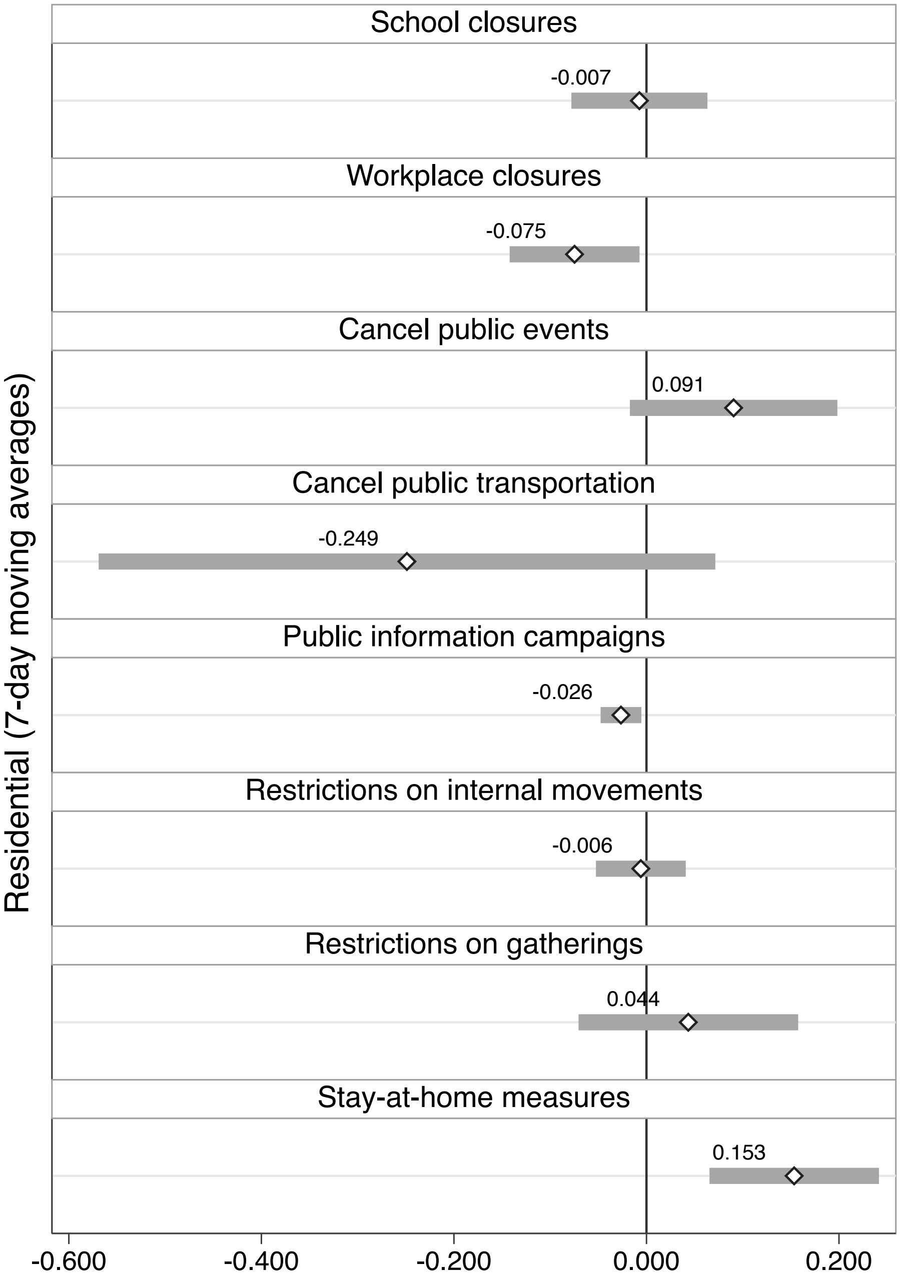


Figure A.4: Disaggregated policy responses, controlling for subnational policies. All Two-Stage Least Squares regression models include our baseline controls together with subnational policy dummies. Coefficients are represented by open dots and their respective numbers. Bars are 95% confidence intervals, calculated with Driscoll-Kraay standard errors.

Table A.21: Summary of Previous Research

| Study | Sample | Method | Main results |
|---|---|---|---|
| Bartik et al. [3] | Survey of more than 5,800 business in the U.S. between March 28 and April 4, 2020. | Descriptive statistics | 41.3% of businesses were temporarily closed because of COVID-19; 1.8% reported that they were permanently closed because of the pandemic; 1.3% reported that they were temporarily closed for other reasons; 55.5% reported that they were still operational. |
| Bendavid et al. [4] | Observational data from subnational regions from England, France, Germany, Iran, Italy, Netherlands, Spain, South Korea, Sweden and the U.S. | First difference models with fixed effects | Implementing mandatory stay-at-home and business closures was associated with significant reductions in case growth of COVID-19 in 9 out of 10 study countries (Spain had a nonsignificant effect). |
| Bonaccorsi et al. [6] | Observational data (including Facebook's mobility data) from Italy. | Network and (quantile and OLS) regression analysis. | Reduction in connectivity tends to be stronger for municipalities with low average individual income and high income inequality. At the same time, mobility restrictions have a higher impact on municipalities with higher fiscal capacity. |
| Chan et al. [8] | Observational data (including Google's mobility data) from 58 countries. | Random effects linear model | Risk-taking behavior is positively associated with the change in visitation retail and recreation places ($\beta$ = 2.873, p = 0.015), and parks ($\beta$ = 7.667, p = 0.003). There is no relationship between risk preference and change in mobility to grocery and pharmacy ($\beta$ = - 0.481, p = 0.650), transit stations ($\beta$ = 1.352, p = 0.317), workplaces ($\beta$ = 0.306, p = 0.718) and residential areas ($\beta$ = - 0.241, p = 0.519). |
| Clinton et al. [10] | 1,135,638 survey respondents between 4 April 2020 and 7 September 2020, and observational data, including Google's mobility data. Both samples are from the U.S. | OLS fixed effects regressions | Partisanship is 27 times more important than the local incidence of COVID-19 in explaining mobility. Moreover, all else equal, Democrats are 13.1% less likely to be socially mobile over time compared to independents, while Republicans are 27.8% more likely to be mobile. |
| Galeazzi et al. [14] | Geolocalized data from 13 M Facebook users in France, Italy, and the UK. | Network analysis | Areas showing higher resilience to mobility disruptions are those where GDP per capita and population density are high. |
| Grossman et al. [16] | Sample of mobile phones users (from Safegraph) in 3,100 counties in the United States during March 2020, county-level partisan preferences, information about the political affiliation of state governors, and the timing of their communications about COVID-19 prevention. | Event study and regression analysis | Governors' recommendations for residents to stay at home had a positive effect on time spent at home, above and beyond the effect of state orders requiring nonessential workers to stay home. A governor's recommendation that residents stay at home increased median time spent at home by 10.4 min per day (or a 3.4% increase) compared with the immediate period before the recommendation was issued. |

*Notes*: The table provides information for samples, methods, and results from the COVID-19 literature (most) related to our study.

Summary of Previous Research — *Continuation*

| **Study** | **Sample** | **Method** | **Main results** |
|---|---|---|---|
| Kochanczyk and Lipniack [18] | Observational data (including Google's mobility data) of 25 highly developed countries and 10 US states. | Pareto-based evaluation | As long as epidemic suppression is the aim, the trade-off between the death toll and economic loss is illusory: high death toll correlates with deep and long-lasting lockdown causing a severe economic downturn. |
| Koren and Petö [19] | Sample of mobile phones users (from Safegraph) in the U.S. between February and May 2020. | Formal model and regression analysis | Employment losses have been largest in sectors that rely heavily on customer contact and where these contacts dropped the most: retail, hotels and restaurants, arts and entertainment and schools. |
| Pulejo and Querubín [23] | Observational data of 65 countries with any constitutionalterm limits on the head of government included in the OxCGRT dataset as of May 29, 2020. | OLS regressions | Incumbents who can run for re-election implement less stringent restrictions when the election is closer in time. The effect is driven by measures more likely to have a negative economic impact. |
| Sheridan et al. [27] | Bank account data from 860,000 active customers of the Danske Bank across Denmark and Sweden. | Natural experiment and regression analysis | Aggregate spending dropped by around 25% in Sweden and, as a result of the shutdown, by 4 additional percentage points in Denmark. This suggests that most of the economic contraction is caused by the virus itself and occurs regardless of social distancing laws. |
| Weill et al. [31] | Observational data (SafeGraph, Google, and Place IQ) of mobility measures and state-level emergency declarations for January–April 2020 in the U.S. | Event study | Wealthy areas went from most mobile before the pandemic to least mobile, while, for multiple measures, the poorest areas went from least mobile to most. |
| Wellenius et al. [32] | Observational data (including Google's mobility data) from the U.S. | Regression discontinuity and multivareable regressions | State-level emergency declarations resulted in a 9.9% reduction in time spent away from places of residence. Social distancing policies resulted in an additional 24.5% reduction in mobility the following week, and subsequent shelter-in-place mandates yielded an additional 29.0% reduction. Decreases in mobility were associated with substantial reductions in case growth two to four weeks later. |
| Xiong et al. [35] | Observational data (including Google's mobility data) at each U.S. county from March 1 to June 9, 2020. | SEM with dynamic panel and time-varying coefficients | External travel to other counties decreased by 35% soon after the nation entered the emergency situation, but recovered rapidly during the partial reopening phase. They also find a positive relationship between mobility inflow and the number of infections during the COVID-19 onset. This relationship is found to be increasingly stronger in partially reopened regions. |

*Notes*: The table provides information for samples, methods, and results from the COVID-19 literature (most) related to our study.

## References

[1] Angrist, Joshua D and Jörn-Steffen Pischke. 2008. *Mostly Harmless Econometrics: An Empiricist's Companion*. Princeton University Press.

[2] Apple. 2020. "Mobility Trend Reports." https://covid19.apple.com/mobility.

[3] Bartik, Alexander W, Marianne Bertrand, Zoe Cullen, Edward L Glaeser, Michael Luca and Christopher Stanton. 2020. "The impact of COVID-19 on small business outcomes and expectations." *Proceedings of the national academy of sciences* 117(30):17656–17666.

[4] Bendavid, Eran, Christopher Oh, Jay Bhattacharya and John PA Ioannidis. 2021. "Assessing mandatory stay-at-home and business closure effects on the spread of COVID-19." *European journal of clinical investigation* 51(4):e13484.

[5] Bolt, Jutta, Robert Inklaar, Herman de Jong and Jan Luiten van Zanden. 2018. "Maddison Project Database 2018." *Groningen Growth and Development Centre* .

[6] Bonaccorsi, Giovanni, Francesco Pierri, Matteo Cinelli, Andrea Flori, Alessandro Galeazzi, Francesco Porcelli, Ana Lucia Schmidt, Carlo Michele Valensise, Antonio Scala, Walter Quattrociocchi et al. 2020. "Economic and social consequences of human mobility restrictions under COVID-19." *Proceedings of the National Academy of Sciences* 117(27):15530–15535.

[7] Cepaluni, Gabriel, Michael Dorsch and Réka Branyiczki. 2021. "Political regimes and deaths in the early stages of the COVID-19 pandemic." *Journal of Public Finance and Public Choice (forthcoming)* .

[8] Chan, Ho Fai, Ahmed Skali, David A Savage, David Stadelmann and Benno Torgler. 2020. "Risk attitudes and human mobility during the COVID-19 pandemic." *Scientific reports* 10(1):1–13.

[9] Cinelli, Carlos and Chad Hazlett. 2020. "Making sense of sensitivity: Extending omitted variable bias." *Journal of the Royal Statistical Society: Series B (Statistical Methodology)* 82(1):39–67.

[10] Clinton, Joshua, Jon Cohen, John Lapinski and Marc Trussler. 2021. "Partisan pandemic: How partisanship and public health concerns affect individuals' social mobility during COVID-19." *Science advances* 7(2):eabd7204.

[11] Conley, Timothy G, Christian B Hansen and Peter E Rossi. 2012. "Plausibly exogenous." *Review of Economics and Statistics* 94(1):260–272.

[12] Coppedge, Michael, John Gerring, Carl Henrik Knutsen, Staffan I Lindberg, Jan Teorell, David Altman, Michael Bernhard, M Steven Fish, Adam Glynn, Allen Hicken et al. 2020. "V-Dem Codebook V10.".

[13] Freedom House. 2019. "Freedom in the world 2019: Democracy in retreat. Washington DC: Freedom House.".

[14] Galeazzi, Alessandro, Matteo Cinelli, Giovanni Bonaccorsi, Francesco Pierri, Ana Lucia Schmidt, Antonio Scala, Fabio Pammolli and Walter Quattrociocchi. 2021. "Human mobility in response to COVID-19 in France, Italy and UK." *Scientific reports* 11(1):1–10.

[15] Google LLC. 2020. "Google COVID-19 Community Mobility Reports." https://www.google.com/covid19/mobility/.

[16] Grossman, Guy, Soojong Kim, Jonah M Rexer and Harsha Thirumurthy. 2020. "Political partisanship influences behavioral responses to governors' recommendations for COVID-19 prevention in the United States." *Proceedings of the National Academy of Sciences* 117(39):24144–24153.

[17] Hale, Thomas, Anna Petherick, Toby Phillips and Samuel Webster. 2020. "Oxford COVID-19 Government Response Tracker." *Blavatnik School of Government* .

[18] Kochańczyk, Marek and Tomasz Lipniacki. 2021. "Pareto-based evaluation of national responses to COVID-19 pandemic shows that saving lives and protecting economy are non-trade-off objectives." *Scientific reports* 11(1):1–9.

[19] Koren, Miklós and Rita Pető. 2020. "Business disruptions from social distancing." *Plos one* 15(9):e0239113.

[20] Marshall, Monty G, Ted Robert Gurr and Keith Jaggers. 2019. "Polity IV project: Political regime characteristics and transitions, 1800-2017.".

[21] National Oceanic and Atmospheric Administration. 2020. "Global Surface Summary of the Day – GSOD." https://data.noaa.gov/dataset/dataset/global-surface-summary-of-the-day-gsod.

[22] PRS, Group. 2019. "International Country Risk Guide, Political Risk Services.".

[23] Pulejo, Massimo and Pablo Querubín. 2021. "Electoral concerns reduce restrictive measures during the COVID-19 pandemic." *Journal of Public Economics* 198:104387.

[24] Raleigh, Clionadh, Andrew Linke, Håvard Hegre and Joakim Karlsen. 2010. "Introducing ACLED: An Armed Conflict Location and Event Dataset: Special Data Feature." *Journal of Peace Research* 47(5):651–660.

[25] Roser, Max, Hannah Ritchie, Esteban Ortiz-Ospina and Joe Hasell. 2020. "Coronavirus Pandemic (COVID-19)." *Our World in Data* . https://ourworldindata.org/coronavirus.

[26] Sebhatu, Abiel, Karl Wennberg, Stefan Arora-Jonsson and Staffan I. Lindberg. 2020. "Explaining the homogeneous diffusion of COVID-19 nonpharmaceutical interventions across heterogeneous countries." *Proc. Natl. Acad. Sci. U.S.A.* 117(35):21201–21208.

[27] Sheridan, Adam, Asger Lau Andersen, Emil Toft Hansen and Niels Johannesen. 2020. "Social distancing laws cause only small losses of economic activity during the COVID-19 pandemic in Scandinavia." *Proceedings of the National Academy of Sciences* 117(34):20468–20473.

[28] Teorell, Jan, Stefan Dahlberg, Soren Holmberg, Bo Rothstein, Natalia Alvarado Pachon and Sofia Axelsson. 2020. "The Quality of Government Standard Dataset, version Jan20, University of Gothenburg.".

[29] The World Factbook, CIA. 2020. "Total Number of Airports by Country." `https://www.cia.gov/the-world-factbook/field/airports/country-comparison`.

[30] Wang, Albert. 2013. "Average Latitude Longitude Countries." `https://github.com/albertyw/avenews/blob/master/old/data/average-latitude-longitude-countries.csv`.

[31] Weill, Joakim A, Matthieu Stigler, Olivier Deschenes and Michael R Springborn. 2020. "Social distancing responses to COVID-19 emergency declarations strongly differentiated by income." *Proceedings of the National Academy of Sciences* 117(33):19658–19660.

[32] Wellenius, Gregory A, Swapnil Vispute, Valeria Espinosa, Alex Fabrikant, Thomas C Tsai, Jonathan Hennessy, Andrew Dai, Brian Williams, Krishna Gadepalli, Adam Boulanger et al. 2021. "Impacts of social distancing policies on mobility and COVID-19 case growth in the US." *Nature communications* 12(1):1–7.

[33] WHO. 2003. "Summary Table of SARS Cases by Country, 1 November 2002-7 August 2003." *Weekly Epidemiological Record* 78(35):310–311.

[34] World Bank. 2016. "World Development Indicators (WDI)." *The World Bank, Washington DC* .

[35] Xiong, Chenfeng, Songhua Hu, Mofeng Yang, Weiyu Luo and Lei Zhang. 2020. "Mobile device data reveal the dynamics in a positive relationship between human mobility and COVID-19 infections." *Proceedings of the National Academy of Sciences* 117(44):27087–27089.
